# Structural basis for safe and efficient energy conversion in a respiratory supercomplex

**DOI:** 10.1038/s41467-022-28179-x

**Published:** 2022-01-27

**Authors:** Wei-Chun Kao, Claire Ortmann de Percin Northumberland, Tat Cheung Cheng, Julio Ortiz, Alexandre Durand, Ottilie von Loeffelholz, Oliver Schilling, Martin L. Biniossek, Bruno P. Klaholz, Carola Hunte

**Affiliations:** 1 Institute of Biochemistry and Molecular Biology, ZBMZ, Faculty of Medicine, University of Freiburg, Freiburg, Germany; 2 Centre for Integrative Biology (CBI), Department of Integrated Structural Biology, IGBMC, CNRS, Inserm, Université de Strasbourg, Illkirch, France; 3 Institute of Genetics and of Molecular and Cellular Biology (IGBMC), Illkirch, France; 4 Centre National de la Recherche Scientifique (CNRS), UMR 7104 Illkirch, France; 5 Institut National de la Santé et de la Recherche Médicale (Inserm), U964 Illkirch, France; 6 Université de Strasbourg, Illkirch, France; 7 Institute of Surgical Pathology, Medical Center – University of Freiburg, Faculty of Medicine, University of Freiburg, Freiburg, Germany; 8 BIOSS Centre for Biological Signalling Studies, University of Freiburg, Freiburg, Germany; 9 Institute of Molecular Medicine and Cell Research, Faculty of Medicine, University of Freiburg, Freiburg, Germany; 10 CIBSS – Centre for Integrative Biological Signalling Studies, University of Freiburg, Freiburg, Germany; 11 Present Address: Ernst Ruska-Centrum für Mikroskopie und Spektroskopie mit Elektronen, Forschungszentrum Jülich GmbH, Jülich, Germany; 12 Present Address: Multiscale Bioimaging Cluster Excellence MBExC, University of Göttingen, Göttingen, Germany; 13 Present Address: Institute of Neuropathology, University Medical Center Göttingen, Göttingen, Germany

**Keywords:** Bioenergetics, Cryoelectron microscopy

## Abstract

Proton-translocating respiratory complexes assemble into supercomplexes that are proposed to increase the efficiency of energy conversion and limit the production of harmful reactive oxygen species during aerobic cellular respiration. Cytochrome *bc* complexes and cytochrome *aa*_3_ oxidases are major drivers of the proton motive force that fuels ATP generation via respiration, but how wasteful electron- and proton transfer is controlled to enhance safety and efficiency in the context of supercomplexes is not known. Here, we address this question with the 2.8 Å resolution cryo-EM structure of the cytochrome *bcc*-*aa*_3_ (III_2_-IV_2_) supercomplex from the actinobacterium *Corynebacterium glutamicum*. Menaquinone, substrate mimics, lycopene, an unexpected Q_c_ site, dioxygen, proton transfer routes, and conformational states of key protonable residues are resolved. Our results show how safe and efficient energy conversion is achieved in a respiratory supercomplex through controlled electron and proton transfer. The structure may guide the rational design of drugs against actinobacteria that cause diphtheria and tuberculosis.

## Abstract (web summary)

Aerobic energy metabolism is driven by proton-pumping respiratory supercomplexes. The study reports the structural basis for energy conversion in such supercomplex. It may aid metabolic engineering and drug design against diphtheria and tuberculosis.

## Introduction

Respiratory chain complexes execute distinct mechanisms to convert energy harnessed from oxidation of food sources into an electrochemical potential gradient to drive adenosine triphosphate synthesis^1^. Cytochrome (cyt) *bc* complexes and cyt *c* oxidases are key contributors^2,3^, which are nearly ubiquitous in aerobic species^1,4,5^. Their association in higher-order assemblies or supercomplexes was described for eukaryotes and prokaryotes and the role of such assemblies for efficient use of substrates and control of oxidative stress is discussed^6–12^. In line with the central role of the two complexes in energy metabolism, their core catalytic subunits are highly conserved across all species^1^. The cyt *bc* complex is a quinol oxidoreductase that translocates protons via the Q cycle mechanism^13^. Its operation is prone to bypass reactions which generate reactive oxygen species (ROS) via a semiquinone radical, resulting in energy waste and deleterious radicals^13^. Unproductive reactions can be triggered through an unbalanced redox-state of the quinone pool, hypoxia, or inherited diseases^14–17^. In cyt *c* oxidases, the key to energy conversion is a coupling of dioxygen reduction at the canonical binuclear centre (BNC) with proton pumping^2^. It requires uptake of protons for dioxygen reduction as well as for proton release^18^. For efficient operation, protons need to be delivered on-demand, their release enabled against an electrochemical potential, and non-productive leaks along the gradient prevented. The underlying mechanism for controlled proton transfer is not well understood^1,2^.

**Fig. 1:**
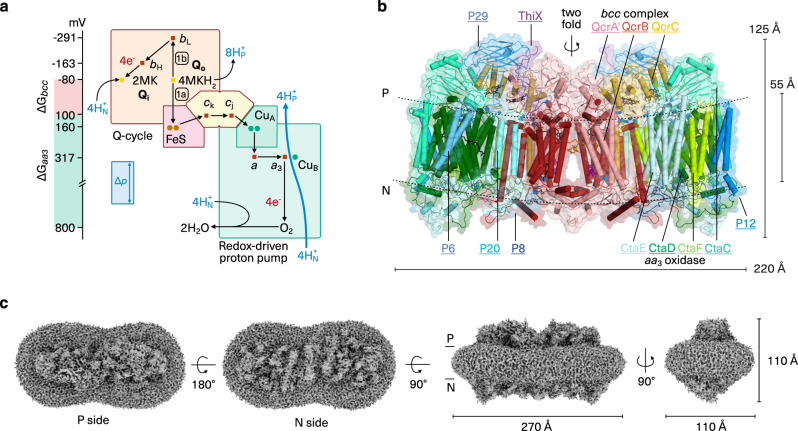
Cyt *bcc*-*aa*_3_ supercomplex. **a** Schematic presentation of energy conversion in the obligate respiratory supercomplex. The cyt *bcc* complex operates a Q cycle with menaquinol oxidation at the Q_o_ site coupled to proton release to the electropositive membrane side (H^+^_P_), and menaquinone reduction at the Q_i_ site with proton uptake from the electronegative membrane side (H^+^_N_), linked through bifurcated electron transfer. The oxidase operates as a redox-driven proton pump. Electron transfer routes are mapped on catalytic subunits with redox-active cofactors: QcrB (haem *b*_L_, haem *b*_H_), QcrA (2Fe-2S cluster, FeS), di-haem QcrC (haem *c*_k_, haem *c*_j_), CtaC (Cu_A_), CtaD (haem *a*, haem *a*_3_, Cu_B_). The net reaction for reducing one dioxygen molecule is shown. Δp denotes the 200 mV proton motive force of *C. glutamicum*^56^. Redox midpoint potentials were taken from the previous study^21^. **b** Cryo-EM structure of cyt *bcc*-*aa*_3_ supercomplex. The atomic model of the homodimer is viewed parallel to the membrane shown in transparent surface and superimposed in cartoon representation. Subunits are colour-coded with matching underlined labels. QcrA crosses the dimer and in homology to the mitochondrial cyt *bc*_1_ complex, the subunit is assigned to that protomer, in which the transmembrane anchor of the catalytic domain is associated. QcrA´ thus denotes the subunit of the other protomer. Cofactors and selected ligands are shown in the ball-and-stick presentation. P and N denote the periplasmic/electropositive and cytosolic/electronegative side of the membrane, respectively. **c** 3D reconstruction of supercomplex with dimensions and detergent micelle. P and N denote the electro-positive and -negative sides of the membrane, respectively. The contour level of the experimental map was set to 3.5 root mean square deviation (rmsd).

The actinobacterial cyt *bcc*-*aa*_3_ supercomplex combines a menaquinol oxidising cyt *bc* complex with an *aa*_3_-type oxidase^19–21^. The energetics of cyt *bc* complexes are adapted to the quinone species^21^. The actinobacterial supercomplex is an obligatory association, as this large bacterial phylum lacks mobile cyt *c*^21^ and both complexes need to interact to enable direct electron transfer between the complexes^20,21^ (Fig. 1a). First structures of the cyt *bcc*-*aa*_3_ supercomplex from *Mycobacterium smegmatis* resolved its overall architecture^22,23^. Here, we report the high-resolution cryo-EM structure of the cyt *bcc*-*aa*_3_ supercomplex from *Corynebacterium glutamicum*, the prototype for such a complex. The resolved catalytic position of menaquinol and of two associated proton channels provide the basis for the concerted release of electrons and protons limiting wasteful and deleterious bypass reactions in the cyt *bcc* complex. A lycopene molecule and a previously unknown Q_c_ site occupied by menaquinone are well suited as electron buffer and further minimise the risk of radical formation. The resolved conformational states of three conserved key protonable residues and of haem *a*_3_ propionate δ provide the basis for controlled proton uptake, loading and release and thus for effective proton pumping in cyt *c* oxidases. A dioxygen molecule is resolved in the gas migration channel which is constricted by the gating residue of the D proton channel, suggesting a coordinated oxygen and proton delivery to the active site for catalysis. Our results show how safe and efficient energy conversion is achieved in a respiratory supercomplex. Our study may enable for metabolic engineering of medically and economically important actinobacteria^24^, a highly diverse phylum that includes producers of amino acids and other natural products including clinically used antibiotics, and could foster rational drug design against pathogenic actinobacteria which cause diphtheria and tuberculosis^25^.

## Results

### Overall structure and lipid constituents

We purified the cyt *bcc*-*aa*_3_ supercomplex from *C. glutamicum* (Supplementary Fig. 1) and determined the cryo-EM structure at an average resolution of 2.8 Å and a local map resolution up to 2.5 Å (Fig. 1, Supplementary Figs. 2, 3 and Supplementary Tables 1, 2). The high resolution permitted a detailed structural description including precise geometry of prosthetic groups, ligands, lipids, and ordered solvent molecules. The supercomplex forms a pseudo twofold symmetrical compact rod with a slight curvature in the membrane plane (Fig. 1b). The dimeric cyt *bcc* complex is located in the centre and on both sides, a monomeric cyt *aa*_3_ oxidase is attached. Each supercomplex protomer comprises thirteen subunits, catalytic subunits QcrABC of cyt *bcc* complex, catalytic subunits CtaCDEF of cyt *aa*_3_ oxidase and the six supernumerary subunits P29, P20, P12, P8, P6 and ThiX (Fig. 1b and Supplementary Fig. 4). ThiX, P12, P8 and P6 were identified in the cryo-EM map and were previously not known to be accessory subunits of the *Corynebacterium* supercomplex, for which they are specific (Supplementary Table 3). Assignment of the supernumerary subunits was confirmed by mass spectrometry (Supplementary Table 4). Subunits P29 and P20 have structurally similar counterparts in the mycobacterial supercomplex^22,23^ and were previously biochemically shown as constituents of the *C. glutamicum* supercomplex^19^. Information on ThiX is scarce. Genetic studies showed that *thiX* has a function in the thiamine biosynthetic process^26^, yet the enzymatic activity of the gene product is not known so far. The soluble domain of ThiX was not resolved most likely due to its flexibility. This is akin to the disordered superoxide dismutase subunit of the *M. smegmatis* supercomplex^22,23^, which occupies the equivalent position of ThiX but lacks a homologue in *C. glutamicum*. The catalytic subunits of the *C. glutamicum* supercomplex share high structural homology with those of the *M. smegmatis* supercomplex (root mean square deviation between 0.8 and 1.9 Å, amino acid sequence ranges between 52 and 84%; Supplementary Table 3), whereas the composition and structures of the supernumerary subunits is specific for *C. glutamicum* as compared to *M. smegmatis* with the largest agreement between P29, P20 and LpqE/ PRASF1, respectively (Supplementary Table 3).

**Fig. 2:**
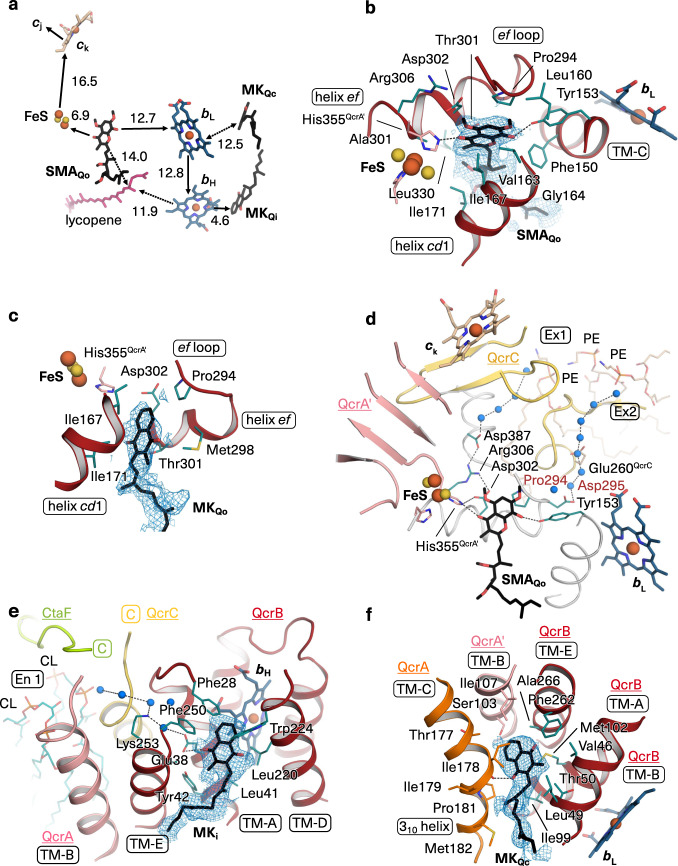
Quinone binding sites and proton transfer pathways in cyt *bcc* complex. **a** Redox-active cofactors, endogenous MK in Q_i_ and unexpected Q_c_ site, stigmatellin (SMA) at Q_o_ site, and lycopene are shown for one protomer. Electron transfer is indicated with arrows and respective distances (edge-to-edge) are given in Å. **b**–**f** Close-up views of quinone binding sites with parts of the structure removed for clarity. Cryo-EM map (blue mesh) is shown for ligands. Subunits are colour-coded with matching underlined labels. QcrA´ crosses the dimer and is associated with the second protomer. Residue labels refer to QcrB if not otherwise indicated. TM denotes transmembrane helices. Annotation of structural elements of all subunits are provided in Supplementary Fig. 6. Iron and sulfur atoms of cofactors are shown in brown and yellow spheres, respectively, water molecules as blue spheres. Dotted lines indicate H-bonds. **b** Transition-state analogue SMA bound at Q_o_ site. Side chains are shown for residues in up to 4 Å distance to the chromone ring. Side chains of QcrB (brown) and QcrA´ (FeS coordinating His355, His335) are shown in green and pink, respectively. **c** Endogenous MK bound at Q_o_ site with side chains shown for residues in up to 4 Å distance to the MK ring. Colour code and labels are used as in **b**. **d** Proton release pathways (Ex1, Ex2) from Q_o_ site to the protein surface mediated by H-bonded protonable residues and water molecules. Q_o_ motif residues Pro294 and Asp295 of QcrB (grey) are labelled in red, other QcrB residues are labelled in black. PE denotes phosphatidylethanolamine. **e** Endogenous MK bound at Q_i_ site. En1 denotes the proton uptake pathway. CL stands for cardiolipin. The boxed and coloured C labels indicate the carboxy-termini of QcrC and CtaF. **f** Endogenous MK bound at Q_c_ site. Map contour level was set to 1.0 rmsd in **b**, **c**, **e** and in **f** to 1.4.

The cryo-EM map revealed N-terminal lipid modifications for ThiX and P29, namely diacyl-glycerol modification and palmitoylation at residues Cys23^ThiX^ and Cys32^P29^, in agreement with amino acid sequence-based predictions (Supplementary Table 2 and Supplementary Fig. 5). ThiX and P29 lack transmembrane helices, but the lipid anchor is bound to the protein surface in the membrane region inserting from the P-side. The N-terminal diacyl-glycerol modification of CtaC at residue Cys21, previously shown by native mass spectrometry^27^, is also resolved in the cryo-EM map and included in the structure. In addition, a total of 41 structural phospholipids including glycophospholipids fill the cavities between cyt *bcc* and cyt *aa*_3_ complexes and at the dimer interface (Supplementary Fig. 5). An acetylated phosphatidylinositol dimannoside (AcPIM_2_) is bound at the intercomplex cavity (Supplementary Fig. 5). A lipomannan (Cg LM-A) fragment (AcPIM_2_-derivative with five mannose units) is located at the entry to the menaquinol oxidation site. The glycolipids lipomannan and AcPIM_2_ are native constituents of the cell envelope of the *Corynebactereae* family that includes pathogenic *M. tuberculosis*^28,29^. They contribute to the low permeability of the cell envelope and the intrinsic tolerance against antibiotics^30^ and can sabotage immunoregulatory responses^31^. In addition, a lycopene molecule is resolved in a narrow tunnel of QcrB close to the Q_o_ site and with access to the intercomplex cavity (Fig. 2a and Supplementary Fig. 5). Spectroscopic analysis confirmed the presence of lycopene in the purified supercomplex (Supplementary Fig. 1d). Lycopene is a biosynthetic intermediate of carotenoids in *C. glutamicum*^32^ and light-inducible carotenoid production to protect from oxidative damage was shown in *C. glutamicum*^33^. The antioxidant nature of the pigment is suited to scavenge radical species^34^. Therefore, lycopene in the quinol oxidation vicinity appears to be a suitable protectant from electron leaks and reactive oxygen species generated through bypass reactions of cyt *bc* complex.

### The menaquinol oxidation site of the cyt *bcc* complex

The key reaction of the Q cycle mechanism of the cyt *bcc* complex is menaquinol oxidation with bifurcated electron transfer which takes place at the Q_o_ site (Fig. 1a). The transition state is a semiquinone and the reaction requires the release of two protons^13,35^. Bifurcated electron transfer without electron leaks requires precise positioning of the substrate with close distances to the electron-accepting cofactors haem *b*_L_ and the Rieske-type iron-sulfur cluster (FeS)^13^. Information on the catalytic position of menaquinol in the Q_o_ site was lacking. The semiquinone transition-state analogue stigmatellin^36^ was previously used for Q_o_ site characterisation of the ubiquinol oxidising mitochondrial cyt *bc*_1_ complex^37^ and was added to the supercomplex prior to cryo-EM grid preparation. The cryo-EM map clearly resolves stigmatellin bound between FeS and haem *b*_L_ with respective distances of 6.9 and 12.7 Å (Fig. 2a, b and Supplementary Fig. 7). The ring plane of stigmatellin is positioned through stacking onto Pro294^QcrB^, which is the first residue of the catalytic Q_o_ motif^5^ (Pro294-Asp295-Val296-Tyr297, c.f. Pro271-Glu272-Trp273-Tyr274 of yeast cyt *b*, Fig. 2b). The orientation within the ring plane is stabilised by multiple non-polar contacts and two hydrogen (H-) bonds. One H-bond is present between the carbonyl oxygen atom of stigmatellin and the FeS ligand His355^QcrA^. The other is present between the hydroxyl moiety of stigmatellin and Tyr153^QcrB^ (Fig. 2a, b), whereas it is H-bonded to Glu272 of the Q_o_ motif in the mitochondrial cyt *bc*_1_ complex^37^ (Supplementary Fig. 7g). The binding close to FeS agrees with the modulation of the EPR spectra of the reduced FeS cluster in the *C. glutamicum* supercomplex^21^. The H-bond to FeS and the proline stacking of stigmatellin are the same in the cyt *bcc* and the mitochondrial cyt *bc*_1_ complex^37^ (Supplementary Fig. 7g) resulting in nearly identical distances to the two electron-accepting cofactors. Taken together, the binding position of the transition state of menaquinol and ubiquinol oxidation and thus the structural basis for electron bifurcation is strictly conserved. In a second structure of the as-isolated supercomplex obtained at an average resolution of 3.1 Å, a co-purified menaquinone molecule was identified close but not fully slid into this catalytic position (Fig. 2c and Supplementary Fig. 7i). The orientation of the ring plane is similar to that of bound stigmatellin but the oxygen atoms are 3.6 Å more distant to the H-bond partners as compared to the respective oxygen atoms of stigmatellin. This menaquinone position likely reflects a product state leaving the active site.

Analysis of amino acid residue conservation for the actinobacterial Q_o_ site indicates that the menaquinol oxidation transition state, as reflected here by stigmatellin bound in the *C. glutamicum* supercomplex, is characteristic for actinobacteria (Supplementary Fig. 8). Out of eleven residues in contact with the chromone ring of stigmatellin, seven are highly conserved in actinobacteria (including Pro294^QcrB^ of the Q_o_ motif and the H-bond providing His355^QcrA^ and Tyr153^QcrB^). Residues providing stabilising contacts in the tail region are species-specific, which might offer opportunities for targeting pathogenic actinobacteria such as *M. tuberculosis*.

### Two pathways for proton release at the Q_o_ site

The two protons generated by menaquinol oxidation need to be released to the electropositive side of the membrane for proton motive force (PMF) generation (Fig. 1a). In mitochondria, the primary proton acceptors of ubiquinol oxidation are the FeS cluster ligand His181 of the extrinsic domain (ED) of the Rieske protein, which undergoes redox-dependent protonation^38^ and most likely the protonable residue of the Q_o_ motif^3,5,37^. These protons could be directly released to the aqueous environment through an opening of the Q_o_ site after quinol oxidation, as the Rieske ED undergoes a large conformational change to deliver electrons from cyt *b* to cyt *c*_1_^37,39,40^. In contrast, the ED of QcrA, the Rieske homologous subunit, is stabilised in the structure via tight interactions with six adjacent subunits including QcrA of the second protomer with a total interface area close to 5000 Å^2^ (Supplementary Fig. 7j–l). Consequently, the locked QcrA ED occludes the Q_o_ site from direct exposure to the positive side of the membrane. We identified two pathways for proton release termed Ex1 and Ex2, which start at the catalytic site marked by stigmatellin (Fig. 2d and Supplementary Table 5). For Ex1 on the FeS cluster side, a file of protonable H-bonded residues starting with the FeS-cluster ligand His355^QcrA^, followed by Asp302^QcrB^, Arg306^QcrB^ and Asp387^QcrB^ and a file of ordered water molecules are well suited for release of protons to the protein surface. For Ex2, the side chain of Asp295^QcrB^, the protonable residue of the catalytic Q_o_ motif in Actinobacteria^5^, and a file of ordered H-bonded water molecules provide the structural basis for proton transfer to the surface at the QcrB:QcrC interface, at which phosphatidylethanolamine (PE) lipid molecules are located. To conclude, the structure indicates that PMF generating menaquinol oxidation is accomplished through bifurcated electron transfer with concomitant bifurcated proton release (Figs. 1, 2d).

### Q_i_ site and unexpected Q_c_ site of cyt *bcc* complex

At the Q_i_ site, the site of menaquinone reduction of cyt *bcc* complex close to the inner (electronegative) side of the membrane (Fig. 1a), a co-purified native substrate (MK_Qi_) was resolved in a cavity formed by QcrB helices A, E and D. It is located in 4.6 Å distance to haem *b*_H_ well suited for rapid electron transfer (Fig. 2e). The position is stabilised by multiple non-polar interactions and a single H-bond between the side chain of Glu38^QcrB^ and one carbonyl-moiety of MK_Qi_. On one side of MK_Qi_, the En1 pathway provides the basis for proton uptake required for the quinone reduction reaction. From a cardiolipin molecule, located in a cavity between cyt *bcc* and cyt *aa*_3_ complex, a file of H-bonded water molecules connects to Lys253^QcrB^ and through a water molecule mediated H-bond to the MK_Qi_ ligand Glu38^QcrB^ (Fig. 2e). Both residues are highly conserved in Actinobacteria (Supplementary Table 5). This resembles the mitochondrial cyt *bc*_1_ complex, in which a comparable connection between Q_i_ site ubiquinone and a surface cardiolipin was shown^41^. In contrast to the mitochondria, in which the Q_i_ site ubiquinone is stabilised by an H-bond to each of the two carbonyl oxygen atoms^37,42,43^, the second carbonyl-moiety of MK_Qi_ has no direct or water molecule mediated contact to a protonable amino acid residue, but it is facing the central void between the cyt *bcc* protomers, the quinone exchange cavity. Thus, the Q_i_ site of the actinobacterial supercomplex is distinct from that of other cyt *bc*_1_ complexes.

Moreover, we identified a previously unknown menaquinone binding site, which we call Q_c_ site referring to its central position (Fig. 2a, f). The ring of the bound co-purified MK_Qc_ is located opposite of the Q_o_ site and in 12.6 Å distance to haem *b*_L_. The isoprenoid tail reaches into the quinone exchange cavity. Indeed, extraction and spectroscopic quantification of menaquinone from the purified supercomplex revealed a ratio of 4.2:1 for menaquinone per supercomplex monomer (Supplementary Fig. 1e), in line with the three resolved menaquinone molecules in the native supercomplex and additional fragmentary map features in the quinone exchange cavity. The binding pocket is formed by QcrB, QcrA and QcrA from the second protomer (QcrA´). It is devoid of protonable residues. Whereas the classical Q_o_-Q_i_ site architecture is a prerequisite for performing the Q cycle, the position of the Q_c_ site implies that electrons can be accepted from haem *b*_L_ (Fig. 2a). Based on the hydrophobic nature of the Q_c_ pocket, its complete lack of ionisable residues and of solvent access, we anticipate that MK_Qc_ could be reduced to a semiquinone anion only. The architecture resembles that of the Q_A_ site in chloroplast photosystem II and in type II anoxygenic photosynthetic reaction centre, in which a semiquinone is stabilised in an apolar environment and functions as a single-electron mediator instead of being an exchangeable substrate^36,44,45^. The Q_c_ site is thus well suited to function for transient storage of surplus electrons, keeping haem *b*_L_ oxidised to aid electron bifurcation and support rapid reduction of semiquinone at the Q_i_ site, in order to limit ROS producing bypass reactions.

### Stable integration of di-haem QcrC

Electrons from menaquinol oxidation are transferred through the di-haem subunit QcrC to the cyt *c* oxidase (Fig. 1a). Conflicting information whether QcrC acts as direct electron wire or as a switch through a conformational change derived from the two published structures of the *M. smegmatis* supercomplex^22,23^. One structure shows the same defined QcrC conformation in both protomers describing QcrC as defined electron wire^22^, whereas the other study described QcrC in an open conformation in one protomer and closed in the other, so that a conformational switch was suggested to regulate electron transfer^23^. For the *C. glutamicum* supercomplex, the entire mature subunit QcrC is well resolved in the cryo-EM map (Fig. 1b and Supplementary Figs. 4, 9). It is composed of a sequential repeat of two small cyt *c* domains (J and K) anchored by a single C-terminal TMH (Supplementary Fig. 9a). The two *c*-type haems face each other with their propionate groups and salt bridges stabilise their interface (Fig. 1b and Supplementary Fig. 9b, c). The structures of the individual domains are realised through stereoisomers of the proline residue next to the methionine ligand of haem *c* (*cis*–Pro103^QcrC^ domain J, *trans*-Pro219^QcrC^ domain K). Importantly, each domain of QcrC is deeply embedded in the supercomplex covered by accessory subunit P29, with respective interface areas greater than 2600 Å^2^ (Supplementary Fig. 9d, e). This strong inter-subunit interaction stands for a static busbar via FeS, haem *c*_k_, haem *c*_j_ and Cu_A_ with respective electron transfer distances of 16.5, 12.7 and 14.4 Å (Supplementary Fig. 9a). The defined distances provide the basis for the rate-limiting electron transfer step between FeS and haem *c*_k_, which will be discussed below.

### Structural basis for rapid proton uptake in the *aa*_3_ oxidase

**Fig. 3:**
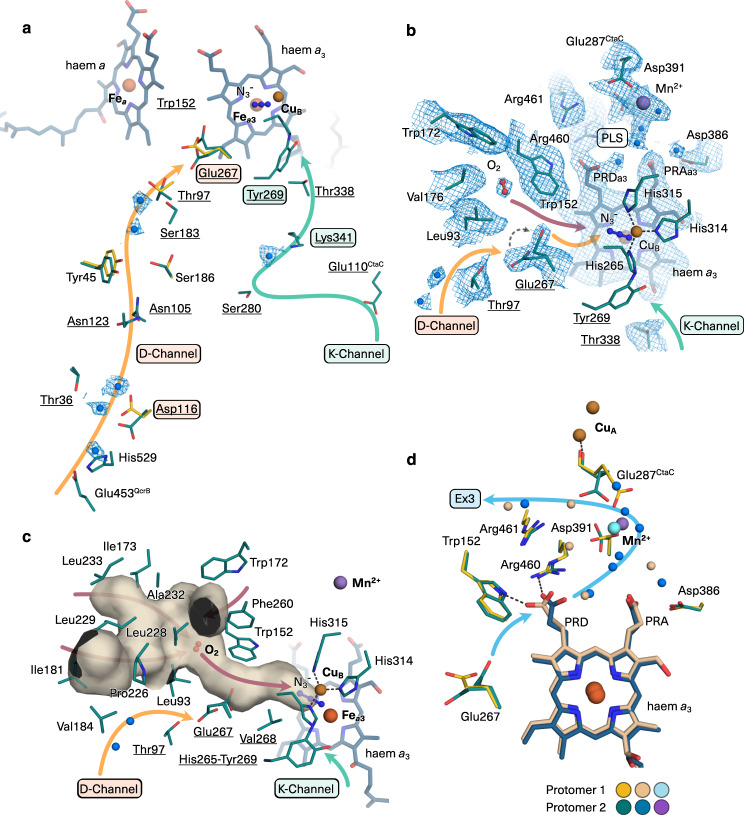
Active sites and proton transfer pathways of cyt *aa*_3_ oxidase. **a** D- and K-proton channels. Constituents of the channels are shown with side chains for protonable and polar residues and the cryo-EM map (blue mesh) for water molecules (blue spheres). Labels of key residues are boxed and conserved residues are underlined. Residues belong to subunit CtaD if not otherwise labelled. Shown in green (carbon atoms) are the elements of protomer 2 (including azide ion (N_3_^−^)). D-channel residues with different side chain conformation in protomer 1 are superimposed (yellow carbon atoms). **b** Close-up view of structure and cryo-EM map (blue mesh) at catalytic centre highlighting molecular oxygen and the alternate conformation of Glu267. The coordination of Cu_B_ is shown with dotted lines, the His265 ligand is covalently bound to Tyr269. PLS denotes the proton loading site. Propionate δ and α of haem *a*_3_ are labelled as PRD_a3_ and PRA_a3_, respectively. **c** Hydrophobic tunnel with dioxygen molecule bound. The two entries at Ile181 and Leu233 face the intercomplex cavity. The tunnel (beige surface) is calculated for Glu267 in down conformation. **d** Proton release route. Superimposition of protomers 1 and 2 highlights conformational states of haem *a*_3_ PRD and of conserved protonable residues. Colour codes differentiate carbon atoms, water molecules and Mn^2+^ of the two protomers as indicated. Ex3 denotes the proton exit pathway. The dotted lines indicate H-bonds. Map contour levels were set to 1.0 rmsd. Supplementary Fig. 10 shows the respective cryo-EM map with distances.

The cyt *aa*_3_ oxidase is a redox-driven proton pump that takes up protons from the electronegative side of the membrane for reduction of molecular oxygen to a water molecule and for pumping protons to the electropositive side against the PMF^2,18^ (Fig. 1a). As the dioxygen reduction site is enclosed in the protein interior, proton uptake channels are required that ensure rapid delivery on-demand. We identified two proton transfer pathways in the structure comprised of two files of protonable and polar residues as well as a series of water molecules (Fig. 3a). This assignment agrees with the canonical K- and D-channels in mitochondrial and proteobacterial A-type cyt *c* oxidases^2,18^. All key residues are conserved (Supplementary Tables 6, 7). The K-channel is marked through the name-giving key residue Lys341^CtaD^ and the channel entry at Glu110^CtaC^. The glutamate side chain points towards the surface of a lipid-filled protein cavity. The characteristic glutamate residue at the end of the D-channel^46^ is Glu267^CtaD^. Typically, an aspartate residue marks the D-channel entry in A-type cyt *aa*_3_ oxidases. This aspartate is key to proton pumping activity^2^ and denotes the D-channel. Asp116^CtaD^ is present at the conserved position, yet it is shielded from the supercomplex surface through a loop of QcrB. Instead, the aspartate side chain is connected to the protein surface through an H-bond path of an ordered water molecule and protonable residues His529^CtaD^ and Glu453^QcrB^, which are both highly conserved in Actinobacteria (Fig. 3a and Supplementary Table 6). Glu453^QcrB^ is part of a 102 amino acid residue long C-terminal extension of QcrB that is not present in mitochondrial cyt *b* (Supplementary Fig. 6). This extension is structurally well defined and docks onto the cyt *aa*_3_ oxidase surface with a total interface area of 1482 Å^2^ with CtaDEF. Phylogenetic sequence analysis showed that the C-terminal extension of QcrB is characteristic for Actinobacteria^5^. Based on structural and phylogenetic data, we suggest an actinobacterial supercomplex specific entry to the oxidase D-channel through the cyt *bcc* complex.

The function of the D-channel in *aa*_3_ oxidases is to take up protons for oxygen reduction and pumping^2^. Molecular dynamic simulations showed that the conserved glutamate residue at the end of the D-channel exhibits protonation-state-dependent conformational changes in bovine cyt *c* oxidase, and it was suggested as gating residue^46^. Here we provide the so far lacking experimental evidence for conformational changes of this gating glutamate. Multiple conformations of Glu267^CtaD^ were revealed taking advantage of the asymmetric reconstruction of the cryo-EM map at high resolution (Fig. 3a). In one protomer, the side chain of Glu267^CtaD^ is present in alternate conformations, one conformation points upwards in the direction of the dioxygen reduction site and the other downwards into the channel towards Thr97^CtaD^. In the other protomer, the side chain of Glu267^CtaD^ is present in a single intermediate conformation. Notably, the conformation of Glu267^CtaD^ at the channel end coincides with defined conformations of Asp116^CtaD^ close to the channel entry (Fig. 3a). With Asp116^CtaD^ pointing towards the channel entry, Glu267^CtaD^ was resolved in alternate conformation. With the aspartate side chain pointing inwards, the glutamate side chain is present in intermediate conformation. This indicates the propagation of conformational changes in the D-channel through a tightly coupled H-bond network providing the structural basis for rapid delivery of protons to the catalytic centre of the oxidase.

### Oxygen delivery to the active site of the *aa*_3_ oxidase

In cyt *c* oxidases, dioxygen reduction takes place at the binuclear centre (BNC) comprised of Cu_B_ and the iron Fe_a3_. This catalytic site is resolved in detail in the supercomplex oxidase with azide bound at the BNC. Azide is known as a potent inhibitor of cyt *c* oxidases^47^. Spectroscopic and structural characterisation with bovine cyt *c* oxidase showed multiple azide binding positions at the dioxygen reduction site^47,48^. In the supercomplex structure, azide ions are resolved in the centre between Cu_B_ and Fe_a3_ in protomer 1 (Supplementary Fig. 10c) and closer to Cu_B_ in protomer 2 (Supplementary Fig. 10d). The coordination of Fe_a3_ and Cu_B_ is consistent in both protomers. Cu_B_ is ligated by three strictly conserved histidine residues (His314^CtaD^, His315^CtaD^, and His265^CtaD^), each with a bond length of 2.0 Å (Supplementary Fig. 10b). The tyrosyl group of Tyr269^CtaD^ is covalently bonded to the Cu_B_ ligand His265^CtaD^ (Supplementary Fig. 10b), which is characteristic for *aa*_3_ oxidases^2^.

Dioxygen needs to be delivered to the active site. Hydrophobic tunnels suitable for oxygen uptake were identified in X-ray structures of cyt *c* oxidases, their assignment were supported by xenon-labelling experiments and computational studies^2^. So far, dioxygen was not identified in such a channel. Here, the cryo-EM map reveals the position of a dioxygen molecule in a pocket formed by side chains of non-polar amino acid residues of CtaD (Trp152, Trp172, Val176 and Leu93) (Fig. 3b). The assignment of dioxygen, as opposed to a water molecule, is supported by the hydrophobic environment and the lack of stabilising hydrogen bonds, which are required for a structurally resolved water molecule. This dioxygen position resembles the equivalent position of a xenon atom in the structure of *ba*_3_ oxidase from *Thermus thermophilus*^49^. Xenon has a similar van der Waals diameter as dioxygen and xenon gas was used in that study to probe hydrophobic cavities suitable for oxygen. We mapped a hydrophobic gas migration tunnel suitable for oxygen uptake in the supercomplex structure. The tunnel is formed by non-polar and aromatic residues (Fig. 3c). It has three entry points at the oxidase surface oriented towards the cyt *bcc* complex (Fig. 3c and Supplementary Fig. 10g) and extends as a narrow tunnel to the dioxygen reduction site (Fig. 3c). The upward orientation of Glu267^CtaD^, the gating glutamate at the end of the D-channel narrows the tunnel and is likely to influence dioxygen delivery to the oxygen reduction site (Fig. 3c and Supplementary Fig. 10g).

### Controlled proton release in the *aa*_3_ oxidase

Essential for proton pumping, the key bioenergetic function of such oxidases is the release of protons to the electropositive membrane side. The nature of the ‘pump site’, that means how protons are uploaded into the exit route, is not fully understood^2^. Computational and spectroscopic studies suggested an involvement of haem propionates and water molecules in the vicinity^50,51^. Here, the structure of the supercomplex oxidase sampled conformational states of protonable structural elements suitable for proton pumping and release. Thanks to cryo-EM image processing without imposing twofold symmetry, the propionate δ of haem *a*_3_ (PRD) could be resolved in two different conformations in the protomers (Fig. 3d and Supplementary Fig. 10e, f). Oriented inwards towards the gating Glu267^CtaD^ of the D-channel, the PRD is in a position suitable for taking up a proton delivered from that channel. Oriented outwards with the closest contact to the guanidino group of Arg460^CtaD^, it can load the proton into an H-bond network for proton release. Notably, the different PRD conformations coincide with altered water molecule positions and side chain positions of Asp391^CtaD^ and the Cu_A_-ligand Glu287^CtaC^, two conserved residues which are part of an H-bond network in a water molecule filled cavity, in which Mn^2+^ is bound (Fig. 3d). The assignment of Mn^2+^ is based on previous EPR spectroscopy data^21^. From Glu287^CtaC^ to the protein surface, a file of protonable residues and water molecules provides a feasible pathway for proton exit (Ex3) (Supplementary Fig. 10h). In conclusion, the structure indicates that PRD serves as an entry point to the proton loading site and the Cu_A_-ligand Glu287^CtaC^ as the exit. Future work needs to show how conformational states are coupled to individual steps of the redox reaction.

## Discussion

**Fig. 4:**
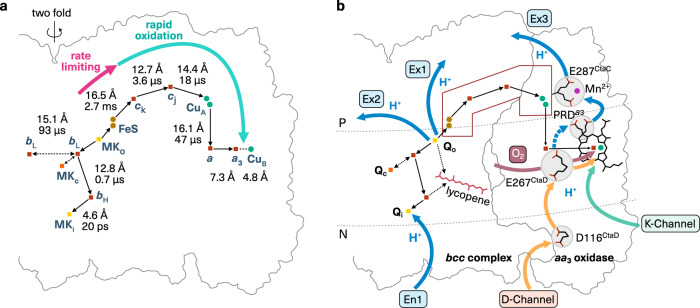
Structural basis for efficient energy conversion in cyt *bcc*-*aa*_3_ supercomplex. Cofactors and substrate molecules of the supercomplex focusing on one protomer are shown with individual coloured icons. The grey line depicts the contour of the supercomplex. **a** Rapid electron transfer within the supercomplex with rate-limiting step between FeS and haem *c*_k_, which is maintained by the fixed conformation of the subunits. Edge-to-edge distances between cofactors and calculated electron transfer rate constants (see Methods) are shown. The menaquinol position in the Q_o_ site (MK_o_) was derived from bound stigmatellin. Detailed distances between MK_c_ and FeS are shown in Fig. 2a. Haem *a*, *a*_3_ and Cu_B_ are strongly coupled in oxidases^100^, thus rates for these electron transfer steps were omitted. The distance between the two haem *b*_L_ may facilitate intermonomer electron transfer as described for cyt *bc*_1_ complex^108^. **b** Q cycle of cyt *bcc* complex with fixed QcrA is enabled through two proton (H^+^) exit routes (Ex1, Ex2). QcrA is homologous to the mobile Rieske protein subunit of mitochondrial cyt *bc*_1_ complex. En1 marks the proton uptake pathway to the Q_i_ site. The additional electron reservoir in Q_c_ site and lycopene (pictogram) can protect against the unproductive and deleterious radical formation. A static electron busbar provides a rapid electron transfer connection between the complexes through QcrA, QcrC and CtaC in fixed conformation (brown-lined box). Different conformational states of key protonable elements (highlighted in grey circles) provide a basis for rapid proton uptake through the D-channel, for proton loading into the exit route via conformational change of propionate δ of haem *a*_3_ (PRD^a3^) and for controlled proton release (Ex3) against proton motive force through coupled conformational states through a Cu_A_ ligand. Dioxygen and proton delivery to the binuclear centre might be coordinated through Glu267^CtaD^ conformational states. The surface of cyt *bcc* complex and *aa*_3_ oxidase is shown as a grey line. The position of the membrane is indicated with dotted lines, with P and N denoting the periplasmic/electropositive and cytosolic/electronegative side, respectively.

The structural characterisation at high resolution sheds light on the mechanism of the cyt *bcc*-*aa*_3_ supercomplex. In contrast to all other *bc* complexes known so far, which enable bifurcated electron transfer with a mobile Rieske protein^1,52^, the homologous subunit QcrA is integrated into the supercomplex as a non-mobile subunit (Fig. 4a, b). Yet, the cyt *bcc* complex fulfils the prerequisite for a Q cycle^1,52^ having a Q_o_ and a Q_i_ site, which were mapped by the transition state analogue stigmatellin and a natively co-purified menaquinone, respectively (Fig. 2b). How does the Q cycle work with a fixed Rieske-type protein and the low potential (−74 mV) menaquinone? The redox potentials of FeS and haem *b*_L_ are well suited for bifurcated electron transfer from menaquinol oxidation (Fig. 1a). They are lower than the ones of the mitochondrial cyt *bc*_1_ complex which oxidises the higher potential (90 mV) ubiquinol^21^ (Fig. 1a). Furthermore, there is experimental evidence that bifurcation in *bc* complexes can take place without Rieske movement, as the kinetic characterisation of the cyt *bc*_1_ complex with immobilised Rieske domain from a *Rhodobacter capsulatus* mutant (plus-two-alanine mutant) still supported bifurcation, whereas the electron transfer to haem *c*_1_ was restricted^53^. The fixed QcrA keeps the Q_o_ site occluded which may minimise the risk of ROS generation, as the reaction intermediate semiquinone can directly reduce dioxygen^17^. Yet, proton release from the closed Q_o_ site is required to fulfil the Q cycle role for PMF generation. The two proton release pathways identified here (Figs. 2d, 4b) provide the basis for the concomitant release of both protons of quinol oxidation ensuring PMF generation and minimising the lifetime of semiquinone. In addition, the identified lycopene (Fig. 2a) and the menaquinone Q_c_ site (Fig. 2f) could store electrons transiently and thus provide further protection from electron leaks and bypass reactions from redox reactions of the supercomplex (Fig. 4b).

The cyt *bcc* complex and its Q cycle are directly linked to the cyt *aa*_3_ oxidase through an electron busbar (Fig. 4b) formed by the stable association and static conformations of QcrA, QcrC and CtaC. Calculated electron transfer rates using the distance between the prosthetic groups (Fig. 4a) and their redox potentials determined previously^21^ (see Methods for detail) reveals rapid electron transfer throughout the supercomplex in µsec and sub-µsec time-scale with one exception in msec time-scale. Calculated rates are in very good agreement with experimental values^20^. Notably, the relatively long distance of 16.5 Å from FeS to haem *c*_k_ with a very small difference in redox potential and thus driving force resulted in the slowest rate of 360 s^−1^ (or 2.7 ms) for the entire electron transfer path (Fig. 4a). It closely matches the experimental turnover number of 210 s^−1^ for quinol:oxidoreductase activity of the supercomplex^20^, thus is justified to be assigned as a rate-limiting step. As the reaction of cyt *c* oxidases can be partially reversible at high ATP concentration and high proton motive force^54,55^, this rate-limiting step can also provide protection of the Q_o_ site from unproductive back reactions within the supercomplex. One should note that the direct coupling of cyt *bcc* complex and cyt *aa*_3_ oxidase has the advantage that the supercomplex exploits the full free energy difference from menaquinol oxidation to dioxygen reduction (ΔG*bcc*-*aa*_3_ = −880 mV, Fig. 1a) under the physiological membrane potential of 200 mV^56^, compensating the lack of a free cyt *c* pool. In mitochondria, the redox-poise of cyt *c* drives the forward reaction by the law of mass action^57^.

Whereas the cyt *bcc* complex shows adaptations to the low potential substrate, the cyt *aa*_3_ oxidase is structurally highly homologous to other cyt *c* oxidases (Supplementary Fig. 6), for instance to the bovine complex or to that of *Paraccocus denitrificans*^58,59^. Therefore, the mechanism of the cyt *aa*_3_ oxidase should be highly conserved among species. The *bcc*-*aa*_3_ branch of the *C. glutamicum* respiratory chain was shown to translocate three protons per electron transferred thus generating proton motive force^60^. This ratio fits two protons being translocated by the Q cycle and one proton being pumped by the oxidase. In line with these considerations, we identified oxidase-typical K- and D-channels for proton uptake^59^ comprised of water molecules and conserved protonable residues. Notably, the D-channel extends through the cyt *bcc* complex emphasising the full integration of both complexes in one functional unit. Moreover, the cryo-EM structure obtained without symmetrical restraints samples in the cyt *c* oxidase a series of conformational states of key protonable residues and moieties which provides unprecedented insights in the structural basis for controlled proton translocation (Fig. 4b). First, conformational changes at D-channel entry and exit indicate a coupled H-bond network. Such a network is ideal for rapid proton delivery through a Grotthuss-type mechanism^1,61^. Second, the conformational change of haem *a*_3_ propionate PRD provides the basis for accepting protons from the D-channel and handing it over to the release route. This finding resolves the open question of the nature of the pump site^2,51^. And third, further release of protons can be facilitated through a conformational change of the protonable Cu_A_ ligand Glu287^CtaC^ suggesting that there is also a coupled H-bond network on the release side. This would be advantageous to prevent unproductive backflow of protons^57^. The described conformational states of Glu267^CtaD^ affect the confluence of D-channel and oxygen channel suggesting the coordination of oxygen and proton delivery to the BNC for an effective turnover. Taken together, the structure of the prototype of the cyt *bcc*-*aa*_3_ supercomplex shows in detail the structural and mechanistic basis of a safe and efficient bioenergetic machinery operating as one unit.

## Methods

### Protein purification and characterisation

*C. glutamicum* cells were cultured and cyt *bcc*–*aa*_3_ supercomplex purified with an optimised purification method based on our previous work^21^. The purification buffer used was Tris-HCl, pH 7.5 with 0.025% dodecyl β-d-maltoside (DDM) and 2 mM MgSO_4_ unless noted. Membranes were solubilized adding 1% DDM. After 1 h of incubation at 4 °C the insoluble material was removed by ultracentrifugation at 186,500 × *g* for 45 min. The supernatant was loaded on a DEAE Sepharose FF column pre-equilibrated with buffer containing 170 mM NaCl, followed by applying the same solution until the absorbance at 415 nm was less than 10 mAU. The supercomplex was eluted with buffer containing 210 mM NaCl and concentrated by ultracentrifugation at 135,700 × *g* for 2 h. The sample was loaded on a size exclusion column (TSKgel 4000 SW) equilibrated with a buffer containing 250 mM NaCl. The first two fractions were collected and concentrated using ultracentrifugation at 135,700 × *g* for 3 h. The purified supercomplex was quantified spectroscopically using the difference spectrum of the sodium dithionite reduced minus potassium ferricyanide oxidised supercomplex at room temperature^21^. The quinol oxidase: dioxygen reductase activity of the purified supercomplex was performed^19^ with dimethyl naphthoquinol which was prepared freshly before use. Dimethyl naphthoquinone (20 mM in ethanol) was reduced by a few grains of sodium borohydride and neutralised by a few drops of 3 N HCl. The activity assay was performed using a Clark-type oxygen electrode (Rank Brothers Digital Model 10) with 100 μM dimethyl naphthoquinol in the reaction buffer (Tris-HCl, pH 7.5, 100 mM NaCl, 2 mM MgSO_4_, 0.025% DDM) to record the initial autooxidation rate of quinol. Purified supercomplex was then added (10 nM) for recording the enzymatic oxygen consumption activity. A 12% NuPAGE Bis-Tris gel was used for SDS-PAGE analysis. The purified supercomplex was subjected to menaquinone extraction^62,63^. About 1 mg of the purified supercomplex was added to 5 ml of 60% (v/v) methanol in petroleum ether (bp 40–60 °C) and mixed thoroughly in a gas-tight vessel. After the two phases were separated, the petroleum ether phase was removed, extracted again with 60% (v/v) methanol and separated from the methanol phase. The petroleum ether phase was dried under a nitrogen stream and the extracted menaquinone was dissolved in ethanol with 10 mM HCl and quantified spectroscopically^64^. All the measurements were performed with four replicates.

### Electron microscopy of the as-isolated (native) sample

About 3 µl of purified supercomplex at a concentration of 10 mg ml^−1^ was applied to a glow-discharged R2/2 300 mesh holey-carbon Quantifoil grid. Subsequently, grids were plunge-frozen in liquid ethane using an FEI Vitrobot (Thermo Fisher Scientific) with a blotting time of 1 s at 95% humidity and 10 °C. Image data were acquired using a spherical aberration (Cs) corrected FEI Titan Krios (Thermo Fisher Scientific) transmission electron microscope operated at an acceleration voltage of 300 kV, equipped with a Gatan GIF energy filter and a K2 Summit direct electron detector camera. Automated data collection was carried out using SerialEM 3.6^65^ in super-resolution mode (super-resolution pixel size = 0.55 Å) at a dose rate of 7.54 e^−^Å^−2^ s^−1^. Six movies were taken per hole, and each movie had a total accumulated exposure of 49.95 e^−^Å^−2^ fractioned into 37 frames. A dataset of 3453 movies was acquired for the native cyt *bcc*–*aa*_3_ supercomplex in a single session using a defocus range between −1.3 and −3.0 µm. Electron microscopy raw frames were Fourier resampled to their physical pixel size of 1.1 Å using the IMOD 4.9.9 software^66^.

### Electron microscopy of the stigmatellin and azide-treated sample

About 3 µl of purified supercomplex at a concentration of 10 mg ml^−1^ with 20 µM stigmatellin (SMA) (Fluka 85865) and 2.5 mM sodium azide (AZI), was applied to a glow-discharged C-flat CF-2/2 Au-50 300 mesh holey-carbon grid. Subsequently, grids were plunge-frozen as for the native sample. Image data were acquired using the aforementioned microscope operated at an acceleration voltage of 300 kV, equipped with an FEI Falcon3 direct electron detector camera. Automated data collection was carried out using EPU 2 (Thermo Fisher Scientific) with a magnification of 75,000 × (pixel size = 0.853 Å) at a dose rate of 1.1 e^−^Å^−2^ s^−1^. Six movies were taken per hole, and each movie had a total accumulated exposure of 40.0 e^−^Å^−2^ fractioned into 30 frames. A dataset of 2833 movies was acquired for the stigmatellin-azide-treated cyt *bcc*–*aa*_3_ supercomplex in a single session using a defocus range between −1.0 and −3.0 µm.

### Cryo-EM data processing

For the native dataset, beam-induced motion and stage drift were corrected by whole frame alignment with a B factor of 1500 Å^2^ and the contrast transfer function (CTF) was corrected using the unblur and ctffind programmes of the cisTEM 1.0.0 beta software package^67^ as well as in RELION 3.1^68,69^. Micrographs with excessively low detected fit resolution estimation (worse than 15 Å) and those which CTF failed to be fitted were excluded from further processing. Particles were automatically picked using crYOLO 1.5.4^70^ from JANNI-denoised^70^ micrographs with the general neural network model trained based on ca. 1000 hand-picked particles. The crYOLO-picked particle positions were imported back to cisTEM using the RELION particle format. Particles images were extracted in cisTEM using a box size of 512 × 512 pixels (native dataset, 1.1 Å pixel^−1^) and one round of 2D classification was used to remove false positives such as ice and classes which did not contain particles. The initial 3D reconstruction was obtained by performing ab initio 3D reconstruction using all particles from the result of a 2D classification of the native dataset, with two starts to reach stable convergence and C2 symmetry was imposed. The 3D reconstruction was refined using 3D Auto Refine of FrealignX with one 3D class and later increased to two 3D classes to allow the well-aligned particles to be consolidated. Manual refinement was carried out using FrealignX in cisTEM with refinement limit iteratively set to 1 Å above the high resolution limit determined according to the Fourier shell correlation (FSC)^71^ using the 0.143 criteria^72^, and two cycles of CTF parameters were refined before finalising the refinement. The dataset was imported to RELION 3.1 for three iterations of Bayesian polishing and CTF refinement. The Bayesian-polished dataset was re-imported to cisTEM for manual refinement (local search starting from refinement limit 8 Å without providing a reference volume). A mask excluding the detergent micelle created by EMAN 2.31^73^ was used to low-pass filter the areas outside the mask by 20 Å with a weight of 1.0 to improve the alignment of the protein in the last step. The best resolution obtained for the native dataset was 3.10 Å (Supplementary Fig. 3).

For the dataset of the SMA-AZI supplemented supercomplex, cryo-EM movies were aligned as described above. Particles were picked using the trained neural network model based on the native dataset using crYOLO 1.5.4 and particles were extracted in cisTEM 1.0.0 beta with the dimension of 580 × 580 pixels. The final map of the native dataset was resampled from 1.1 Å pixel^−1^ to 0.853 Å pixel^−1^ using the SPHIRE 1.3 moon-eliminator pipeline utility^74^ and low-pass filtered to 30 Å to serve as the starting reference of the SMA-AZI dataset, followed by the aforementioned 3D refinement procedure in cisTEM without imposing symmetry (C1) then exported to RELION 3.1. The symmetry was relaxed to C2 using the algorithm implemented in RELION 3.1^75^ and followed by two iterations of CTF refinement and Bayesian polishing. The RELION-refined dataset was re-imported to cisTEM for manual refinement and micelle filtering as described for the native dataset. A final average resolution of 2.8 Å was achieved (Supplementary Fig. 2).

The map of the native dataset was fine-scaled with reference to the SMA-AZI dataset map to calibrate the magnification^76–78^. The calibrated pixel size of the native dataset was 1.08 Å pixel^−1^ and was re-scaled by EMAN 2.31. The final maps of both datasets were further improved by density modification using phenix.resolve_cryo_em^79^ (phenix 1.19.2) using the two half-maps and a soft mask to exclude the bulk-solvent noise whilst retaining the detergent micelle. The density-modified maps were used for model building and refinement.

### Model building and refinement

The initial model was generated using phenix.map_to_model (phenix 1.18.2)^80^ from the cryo-EM map of the native dataset. Manual atomic model building was carried out in the software Coot 0.8.9.2 EL^81^ using sequences of known catalytic subunits. Supernumerary subunits (P12, P8, P6, ThiX) were identified from the de novo built sequence and validated by mass spectrometry. The atomic model was refined using phenix.real_space.refine^82,83^ with B-factor restrained per residue and without rotamer restraints. Occupancy refinement was performed with phenix.real_space_refine 1.20rc1-4392. Geometry restraints of *b*-type haems were adapted to the Crystallography & NMR System (CNS)^84^ topology and parameter files of Lancaster and Michel^36^ to retain the bond length of the porphyrin ring^85^. The geometry restraints of the porphyrin were further modified for *c*-type haems at the vinyl groups to correct the stereochemistry of atoms contributing to the thioether bond when covalently linked to the protein. The geometry restrains of haem *a*_s_ was adapted to the porphyrin geometry of *b*-type haems described above. The peptide-link geometry restraints of the FeS cluster was referenced to that of the 1.5-Å resolution X-ray structure of the isolated Rieske iron-sulfur protein from bovine cyt *bc*_1_ complex pdb 1rie. The peptide-link geometry restraints of *b*-type haems and *c*-type haems *c* were referenced to that of the 1.9-Å resolution X-ray structures of yeast cyt *bc*_1_ complex pdb 3cx5. The peptide-link restraints of *a*-type haems, Cu_A_ and the covalently modified His265-Tyr269 pair were referenced to that of the 1.5-Å resolution X-ray structure of bovine cyt *c* oxidase pdb 5b1a. The peptide-link of Cu_B_ for the structure of the SMA-AZI-bound supercomplex was referenced to pdb 5b1a. For the structure of the native supercomplex, the bond length between the Cε1 atom of His314 and Cu_B_ was restrained to 2.6 Å to obtain the best-refined fit of the imidazole side chain into the density. An Mn^2+^ was modelled to the conserved Mg^2+^/Mn^2+^ site of the cyt *aa*_3_ oxidase near Cu_A_, based on our previous EPR spectroscopy data which confirmed the presence of Mn^2+^ in the supercomplex preparations^21^. Geometry restraints of lipids, menaquinone, lycopene and stigmatellin were calculated using the GRADE server (Global Phasing Limited, http://grade.globalphasing.org). Hydrogen atoms were added to the coordinates using phenix.ready_set to all residues and ligands. The hydrogenated coordinates were subjected to phenix.real_space_refine and manually rebuilt in Coot to improve the geometry. The model geometry was further improved using ISOLDE 1.1^86^, and models were refined again by phenix.real_space_refine for the final optimisation. The refined models were validated using phenix.validation_cryoem^87^. Local resolution was calculated using ResMap 1.1.4^88^. Preferred specimen orientation was validated using the 3D-FSC Server (https://3dfsc.salk.edu)^89^. Model quality was estimated using FSC_work_ and FSC_test_^90^. Figures were prepared using the Open Source PyMol 2.3.0^91^ and UCSF ChimeraX 1.1^92^. Cryo-EM data collection, refinement and validation statistics are shown in Supplementary Table 1.

### Mass spectrometry analysis

Protein solutions were heat-denatured in the presence of an acid-labile surfactant, reduced and alkylated with iodoacetamide, followed by digestion with either trypsin, GluC or chymotrypsin and desalting with C18 reversed-phase solid phase extraction (HyperSep, Thermo Fisher, Langerwehe, Germany). Peptides were analysed by a Q-Exactive plus system (Thermo Scientific) coupled to an Easy nanoLC 1000 with a flow rate of 300 nL/min. Buffer A was 0.1% formic acid, and buffer B was 0.1% formic acid in acetonitrile (water and acetonitrile were at least of HPLC gradient grade quality). A gradient of increasing organic proportion was used for peptide separation (5–25% acetonitrile in 60 min; 25–60% acetonitrile in minutes 60 to 80; 60–90% acetonitrile in minutes 80 to 82). The analytical column was an Acclaim PepMap column (Thermo Scientific) with 2 μM particle sizes, 100-Å pore sizes, length 250 mm, I.D. 50 μM. The mass spectrometer operated in data-dependent mode with a top ten method at a mass range of 300–2000 Da.

LC-MS/MS data in raw format was converted to the mzXML^93^ format, using msconvert (3.0.10385)^94^. For spectrum to sequence assignment, X! Tandem^95^ (Version 2017.02.01) was used. The proteome database consisted of the reference proteome of *C. glutamicum*, downloaded from UniProt on March 4, 2019, appended with the sequences of common contaminants and digestion enzymes. Reversed decoy sequences were used. Search parameters included: pre-cursor mass error of 10 ppm, fragment ion mass tolerance of 20 ppm, tryptic, GluC or chymotryptic specificity with up to one missed cleavage (semi-specific for GluC or chymotrypsin), and cysteine carboxyamidomethylation (57.02 Da). X! Tandem results were further validated by PeptideProphet (implemented in TPP v4.7 rev 0, build 201402281256)^96^ and assembled to proteins using ProteinProphet (implemented in TPP v4.7 rev 0, build 201402281256)^97^.

### Structural and bioinformatic analysis

Default settings of software tools were used unless noted.

Electron transfer rates were calculated from edge-to-edge distance of cofactors using the methods according to Dutton and colleagues^13,98–100^ and the reorganisation energy therein. For haem molecules, distances were calculated from the edge of the conjugated ring^99^. The N-terminal signal peptide and lipoprotein signal peptide were predicted using the SignalP 5.0 server (https://services.healthtech.dtu.dk)^101^. The protein interaction surface area were calculated using the PISA server^102^ (https://www.ebi.ac.uk/pdbe/pisa/). Protein channel and cavity were analysed and visualised using HOLLOW 1.3^103^. The Van der Waals radius of sp2 oxygen^104^ (1.46 Å) was used for the calculation of the oxygen tunnel. Multiple sequence alignment was performed using ClustalOmega 1.2.3^105^ and visualised using JalView 2.10.3b1^106^. The analysis of ligand interaction was performed with LIGPLOT 4.5.3^107^.

### Reporting summary

Further information on research design is available in the Nature Research Reporting Summary linked to this article.

## Supplementary information

Supplementary Information

Reporting Summary

## Data Availability

The cryo-EM density maps are deposited in the Electron Microscopy Data Bank under accession numbers EMD-13976 (SMA-AZI) and EMD-13977 (native). The atomic models of the cryo-EM structures are deposited in the Worldwide Protein Data Bank under accession numbers 7qhm (SMA-AZI) and 7qho (native). All data are available in the main text or supplementary materials. The mass spectrometry data are available at MassIVE as MSV000083873 and at ProteomeXchange as PXD014069. The following coordinates from the protein data bank (pdb) were used to set up the restraints for metal-containing cofactor refinement:1rie, 3cx5, 5b1a. The coordinates of pdb 3cx5 were used for Supplementary Fig. 7h. The coordinates pdb 2a06 and 1be3 were used to calculate the interface area used in Supplementary Fig. 7l. The UniProt (https://www.uniprot.org) Proteome Database was used for LC-MS/MS peptide sequence search. Protein sequences used for multiple sequence alignment for Supplementary Fig. 8 were retrieved from the NCBI Protein Database (https://www.ncbi.nlm.nih.gov/protein).
