# Supplementary material for: Structural basis for safe and efficient energy conversion in a respiratory supercomplex
Source: Nat Commun. 2022 Jan 27;13:545. doi: 10.1038/s41467-022-28179-x (PMC8795186; doi:10.1038/s41467-022-28179-x)
Supplement: Supplementary file 1 — Supplementary Information [file 41467_2022_28179_MOESM1_ESM.pdf]

Supplementary Information

Structural basis for safe and efficient energy conversion in a respiratory supercomplex

Wei-Chun Kao*, Claire Ortmann de Percin Northumberland, Tat Cheung Cheng, Julio Ortiz, Alexandre Durand, Ottilie von Loeffelholz, Oliver Schilling, Martin L. Biniossek, Bruno P. Klaholz, Carola Hunte*

*Correspondence to: wei-chun.kao@biochemie.uni-freiburg.de (W.-C.K.), carola.hunte@biochemie.uni-freiburg.de (C.H.)

This PDF file includes:

Supplementary Figures 1 to 10
Supplementary Tables 1 to 7
Supplementary References 1 to 3

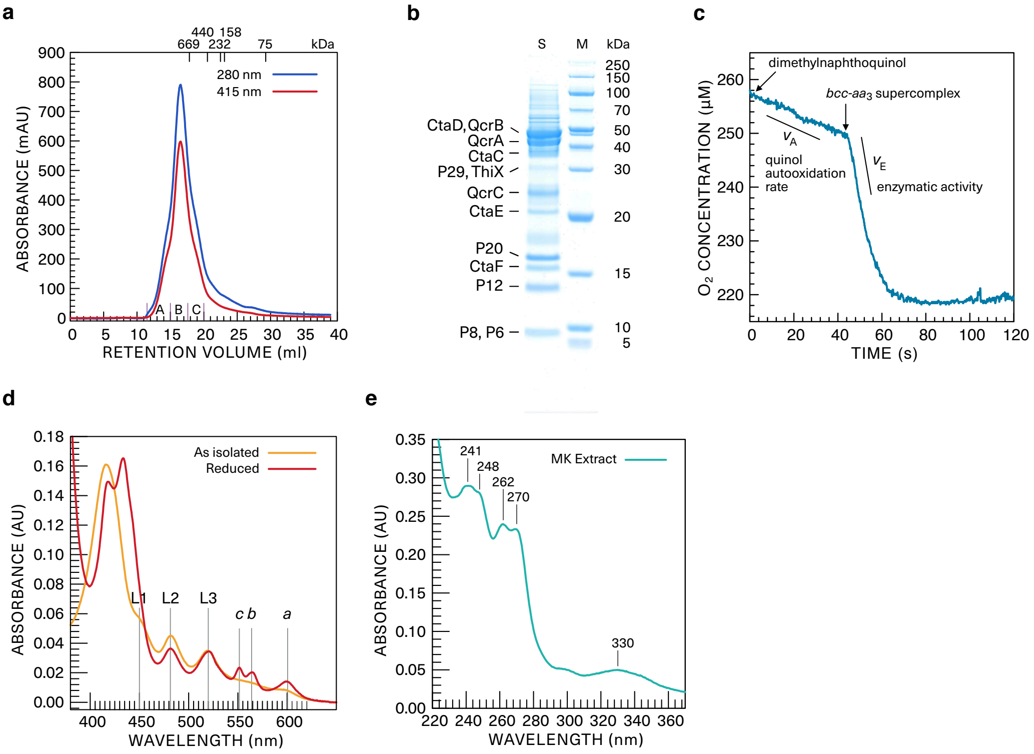

Supplementary Fig. 1 Biochemical characterisation of the cyt *bcc-aa*$_3$ supercomplex from *Corynebacterium glutamicum*. **a** Chromatogram of final purification step by size-exclusion chromatography. The first two fractions were used for cryo-EM single particle analysis. **b** SDS-PAGE analysis of the supercomplex prior to grid preparation (Coomassie-stained gel, supercomplex lane S, molecular weight standard proteins lane M). The experiment was repeated four times and one representative gel image is shown. **c** Enzyme activity of isolated supercomplex. The dimethylnaphthoquinol:dioxygen reductase activity of 112.6 ± 3.4 $e^{-}s^{-1}$ was determined monitoring oxygen consumption. **d** UV-Vis spectroscopic analysis of the as-isolated supercomplex as well as the supercomplex reduced by 0.5 mM sodium dithionite. The three absorption maxima L1 (450 nm), L2 (481 nm), L3 (520 nm) denote the absorbance contributions from lycopene[1]. *a*, *b*, *c*, are the maximum absorbance of *a*-, *b*-, and *c*-type haems at 600 nm, 563 nm and 551 nm, respectively[2]. **e** UV-Vis spectrum of menaquinone (MK) in acidified ethanol extracted from the isolated supercomplex (for details see methods). Supercomplex concentration was determined spectroscopically[2]. The ratio of menaquinone to supercomplex monomer was determined to be 4.2:1.

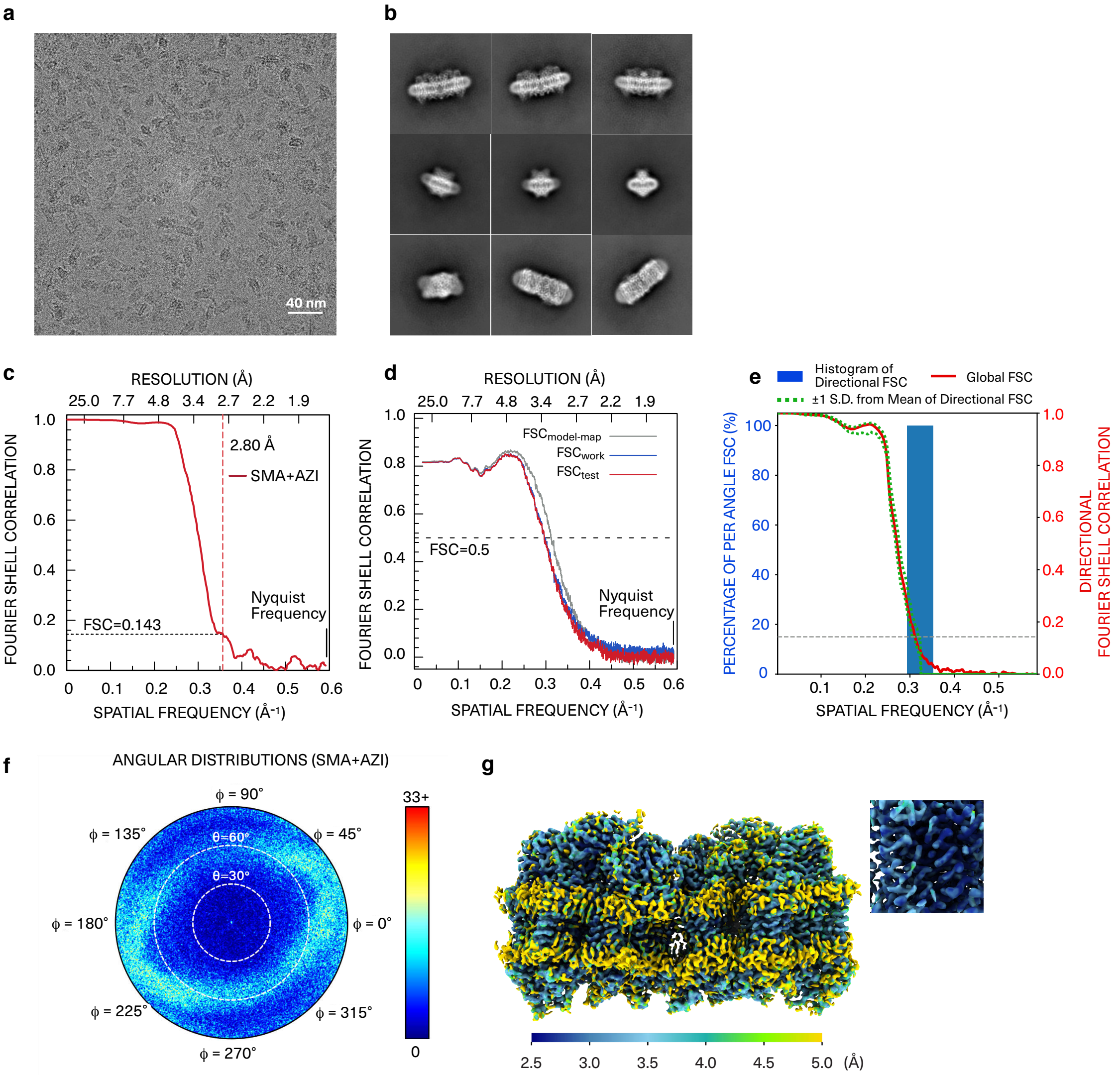

Supplementary Fig. 2 Single particle cryo-EM analysis and validation of the stigmatellin (SMA) and azide (AZI)-treated cyt *bcc-aa*$_3$ supercomplex. a A representative micrograph showing the diverse orientations and homogeneity of the supercomplex particles. A total number of 2833 images was collected from the same grid in one cryo-EM session and 2697 micrographs were used for structure determination (selection criteria see Methods). **b** Selected 2D-class averages of the supercomplex. **c** Fourier shell correlation (FSC) of the final 3D-reconstruction reported by cisTEM 1.0.0. **d** Cross correlation analysis **e** 3D-FSC analysis. **f** Angular distributions of supercomplex. **g** Local resolution estimated by ResMap 1.1.4. In order to visualize the supercomplex only, the detergent micelle was removed for the figure using a mask. The inset shows a selected inner part of the complex at high contour level (7 rmsd).

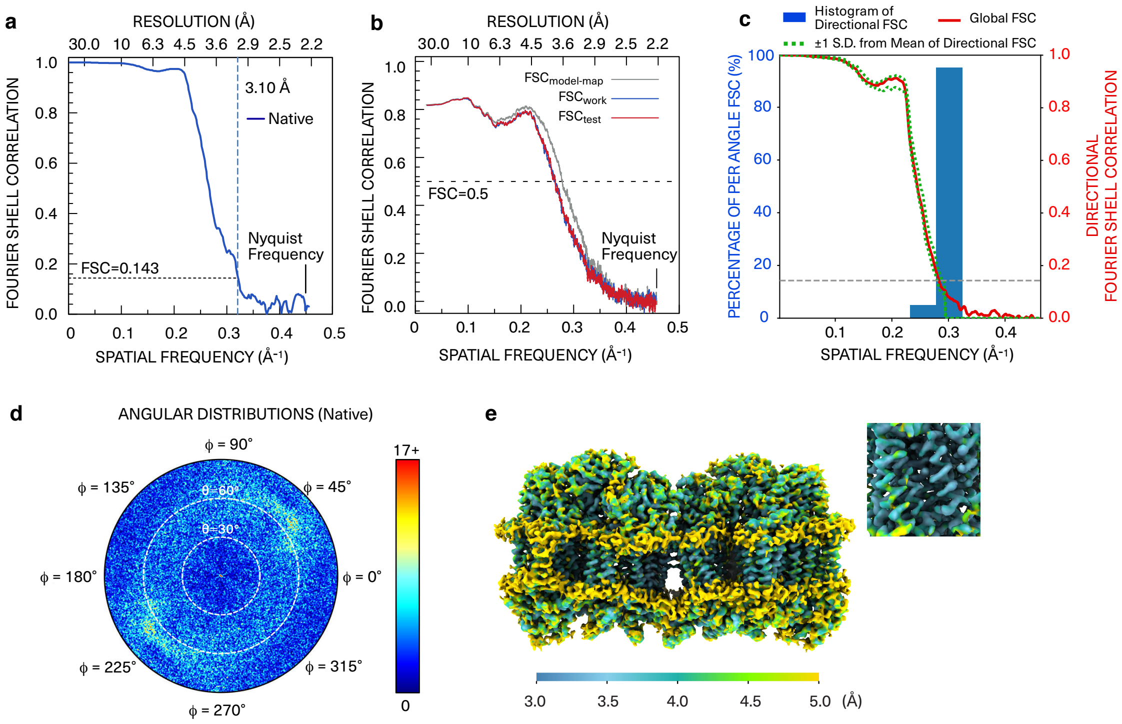

Supplementary Fig. 3 Single particle cryo-EM analysis and validation of the as-isolated cyt *bcc*-*aa*$_3$ supercomplex. a Fourier shell correlation (FSC) of the final 3D-reconstruction reported by cisTEM 1.0.0. **b** Cross correlation analysis. **c** 3D-FSC analysis. **d** Angular distributions of supercomplex. **e** Local resolution estimated by ResMap 1.1.4. In order to visualize the supercomplex only, the detergent micelle was removed for the figure using a mask. The inset shows a selected inner part of the complex at high contour level (7 rmsd).

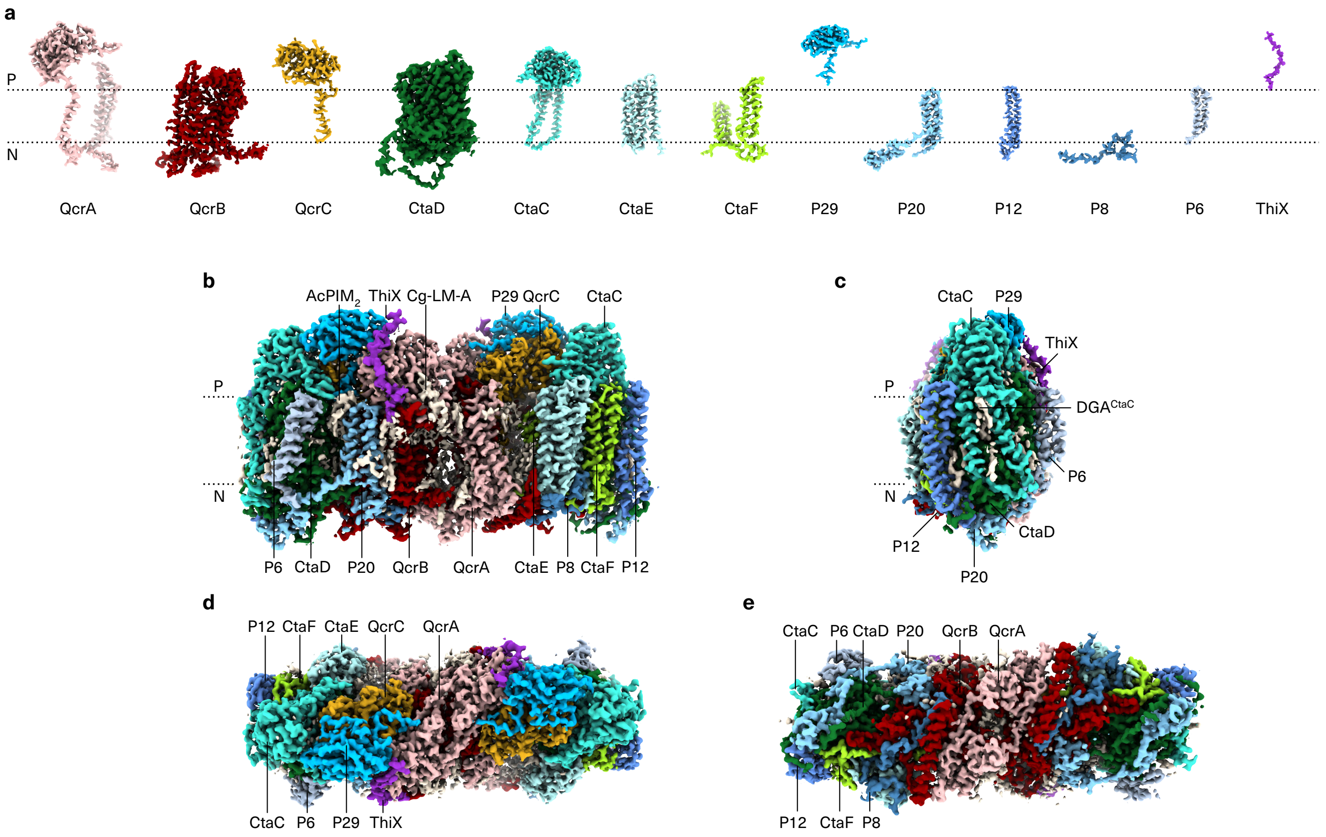

Supplementary Fig. 4 Cryo-EM maps of subunits of *C. glutamicum* cyt *bcc-aa*$_3$ supercomplex. a Cryo-EM map of individual subunits. The approximate membrane boundary (dotted lines) depicts the relative position of the subunits. P and N indicate the electro-positive and -negative sides of the cell membrane. **b-c** The entire complex viewed parallel to the membrane. **d** and **e** show the supercomplex from the P- and N-side of the cell membrane, respectively. The Cryo-EM map is coloured to depict the individual subunits and lipid molecules are shown in beige. Selected lipid molecules (AcPIM$_2$ and Cg-LM-A, the N-terminal covalently linked DGA of CtaC) are highlighted with labels. The contour level of the maps was set to 1.7 rmsd.

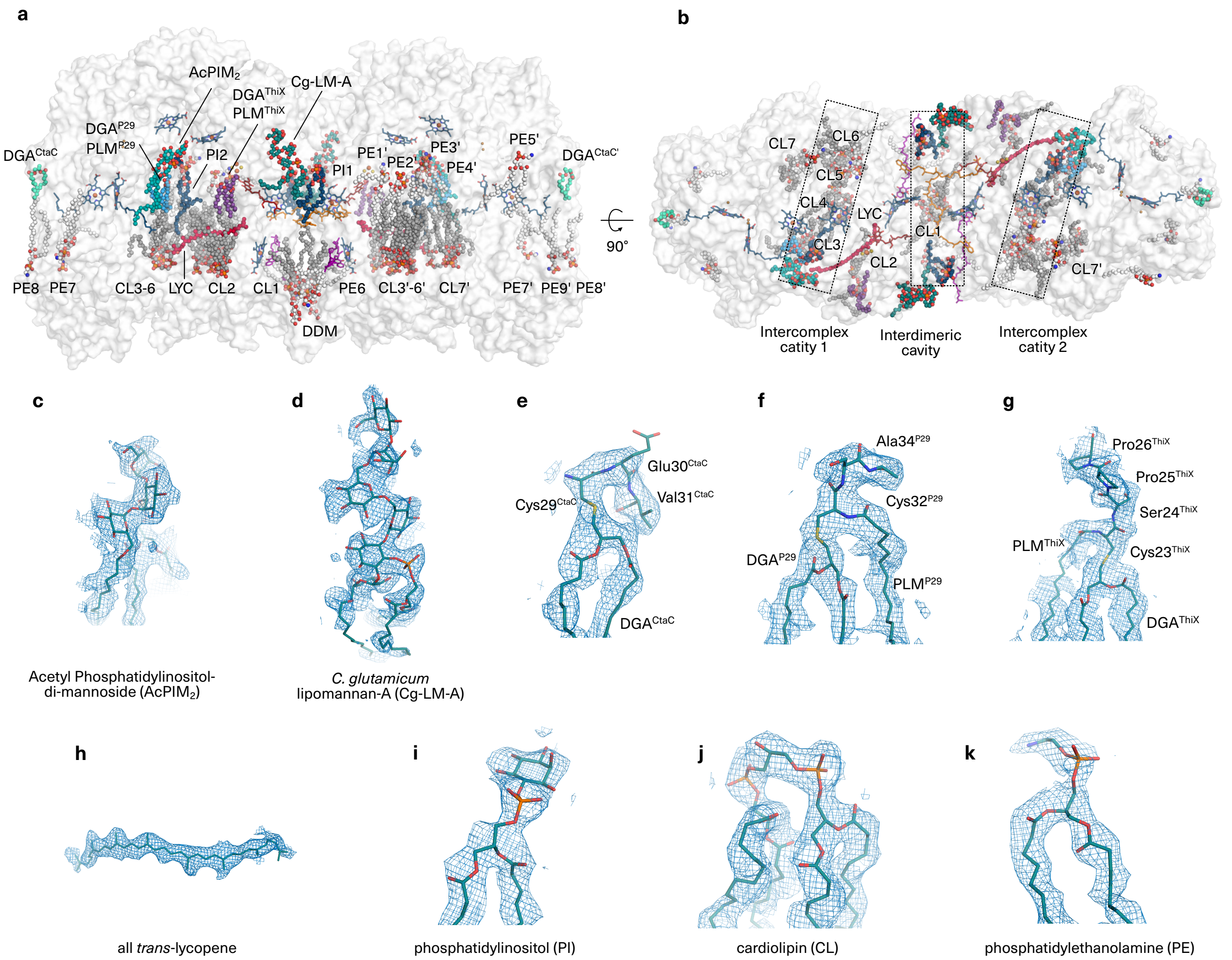

Supplementary Fig. 5 Structural lipids, lipid-modifications and lycopene in cyt *bcc-aa*$_3$ supercomplex structure. **a-b** Lipid molecules are shown in sphere presentation, the supercomplex in transparent surface mode with cofactors and substrates in stick presentation. PE: phosphatidylethanolamine; PI: phosphatidylinositol; CL: cardiolipin; DGA: diacylglycerol; PLM: palmitic acid; LYC: lycopene; AcPIM$_2$: acetylated phosphatidylinositol dimannoside; Cg LM-A: *C. glutamicum* lipomanman-A; DDM: dodecyl β-D-maltoside (detergent). All ligands of the dimeric complex structure are displayed. The numbering of molecules in equivalent position of both protomers are identical, but labeled with a prime (‘) for the second protomer. DGA and PLM are N-terminal modifications of the subunits indicated. **c-d** Structures and cryo-EM map (blue mesh) of glycophospholipids. **e-g** Structures and cryo-EM map (blue mesh) of the N-terminal modifications of CtaC, P29 and ThiX. **h** lycopene **i-k** examples for PI, CL and PE molecules. The contour level of all maps was set to 1.2 rmsd in all panels except to 2.0 rmsd in **g**.

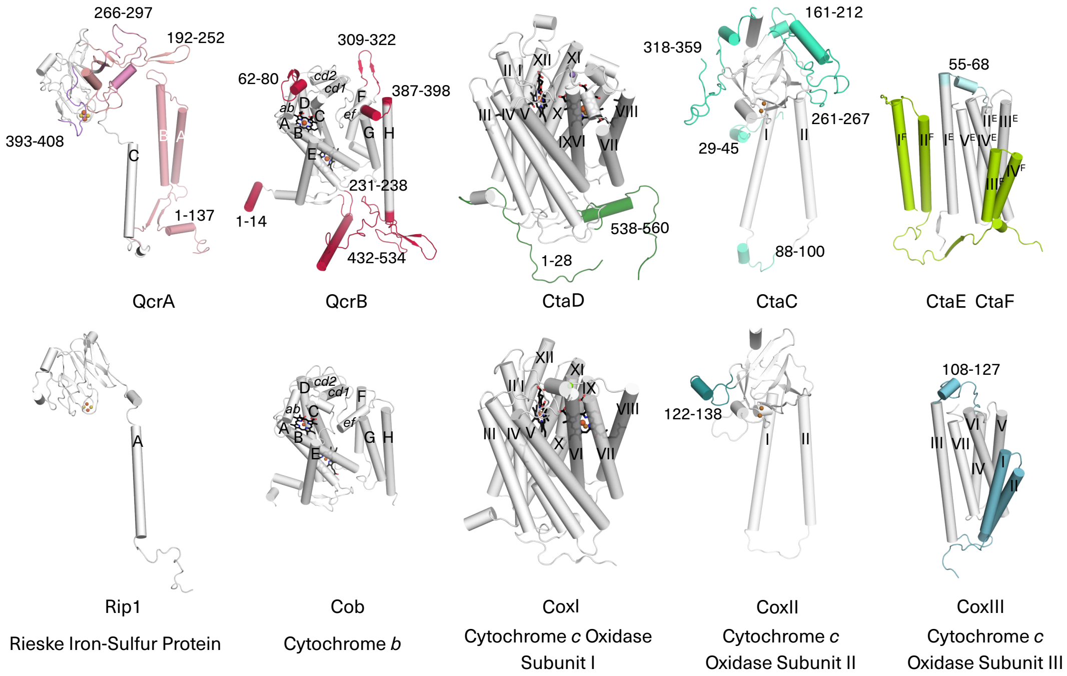

Supplementary Fig. 6 Comparison of cyt *bcc*-*aa*$_3$ supercomplex subunits with homologous subunits of mitochondrial cyt *bc*$_1$ complex and cyt *aa*$_3$ oxidase. Subunits of the supercomplex (upper row) and their mitochondrial counterparts (lower row) are presented in the same orientation in cartoon representation. Conserved structural elements are shown in white; unique parts are highlighted with their residue numbers. Nomenclature of secondary structural elements of the supercomplex follow the conventions of the yeast cyt *bc*$_1$ complex (pdb 3cx5) and bovine cyt *aa*$_3$ oxidase (pdb 5b1a). Note that CtaE is homologous to TMH III-VII of subunit COXIII. THM III-IV of CtaF are homologous to TMH I-II of COXIII.

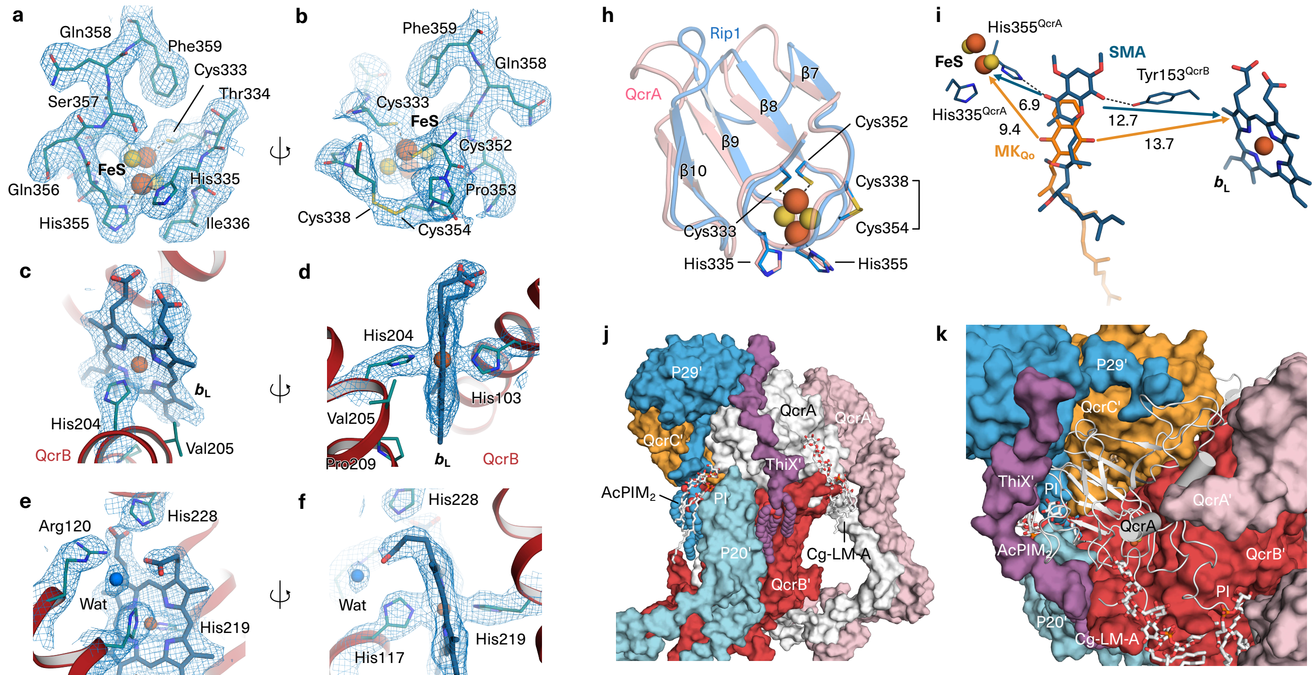

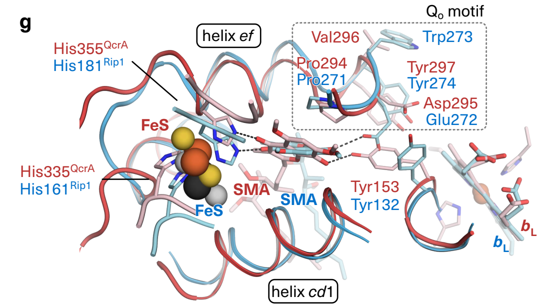

l

Interaction area ($Å^2$) of Rieske iron-sulfur protein (QcrA and Rip1) extrinsic domains (ED)

C. glutamicum		mitochondrial (bovine)		
	QcrA-ED		Rip1-ED (b)	Rip1-ED (c)
QcrB'	2049	Cob'	906	286
QcrC'	912	Cyt1'	0	350
P29'	768	–	–	–
QcrA'-ED	621	Cyt1	53	129
P16'	661	–	–	–
Sum	5011	Sum	959	765

Supplementary Fig. 7 Redox-cofactors of cyt *bcc* complex and interactions of subunit QcrA. a-b Section of QcrA with Rieske iron-sulfur cluster (FeS). Residues shown belong to QcrA . **c-d** Low potential haem b_L embedded in QcrB. Its axial ligand His204 is located on a π-bulge, in which Val205 is in non-bonded contact distance to haem b_L. Residues shown belong to QcrB. **e-f** High potential haem b_H embedded in QcrB. A water molecule (Wat) coordinated by the propionate group and Arg120 is a conserved feature also found in mitochondrial cyt bc_1 complexes. Residues shown belong to QcrB. **g** Comparison of Q_o site with bound stigmatellin (SMA) with that of mitochondrial yeast mitochondrial cyt bc_1 complex. The cyt *b* subunit (blue) was superposed onto the QcrB subunit (brown). Q_o motifs of both complexes are highlighted in the box. The edge-to-edge distances of stigmatellin to FeS and haem b_L are 6.9 and 12.7 Å, respectively, in cyt *bcc* complex, and equivalent distances in the yeast enzyme are 6.8 and 11.4 Å. Dotted lines indicated H-bonds. **h** Comparison of the FeS cluster-binding fold (QcrA, pink) with that in yeast mitochondrial cyt bc_1 complex (Rip1,blue, pdb 3cx5). Residues are numbered according to QcrA. Cys338 and Cys354 form a disulfide bond. **i** Comparison of stigmatellin (SMA) and menaquinone (MK_{Qo}) positions resolved in the Q_o site. Structures of native and stigmatellin-bound supercomplex were superimposed and cofactors and ligand of the latter structure are shown in blue. This includes $Tyr153^{QcrB}$ and $His355^{QcrA}$ with H-bonds (dotted lines) to SMA. MK_{Qo} is shown in orange. Respective edge-to-edge distances are given in Å. **j-k** Integration of QcrA into the complex. The N-terminal lipid modification of P29 and ThiX are shown in spheres. Subunits are shown in surface presentation, those of the other protomer are denoted with a prime ('). Lipids ($AcPIM_2$, PI, Cg-LM-A) are shown in stick presentation. **k** QcrA in cartoon presentation to visualize its interaction to QcrB and QcrC. **l** Interface area ($Å^2$) of the extrinsic domain (ED) of QcrA within the supercomplex and for bovine Rip1 within the cyt bc_1 complex. The mitochondrial Rip1-ED was resolved in different positions in X-ray structures. The "b" position close to cyt *b* (pdb 2a06) and the "c" position close to cyt c_1 (pdb 1be3) were used for calculation. The contour level of maps (blue mesh) was set to 2.2 rmsd in **a** and **b**; to 2.7 rmsd in **c-f**. Iron and sulfur atoms of cofactors are shown in brown and yellow spheres, respectively, water molecules as blue spheres.

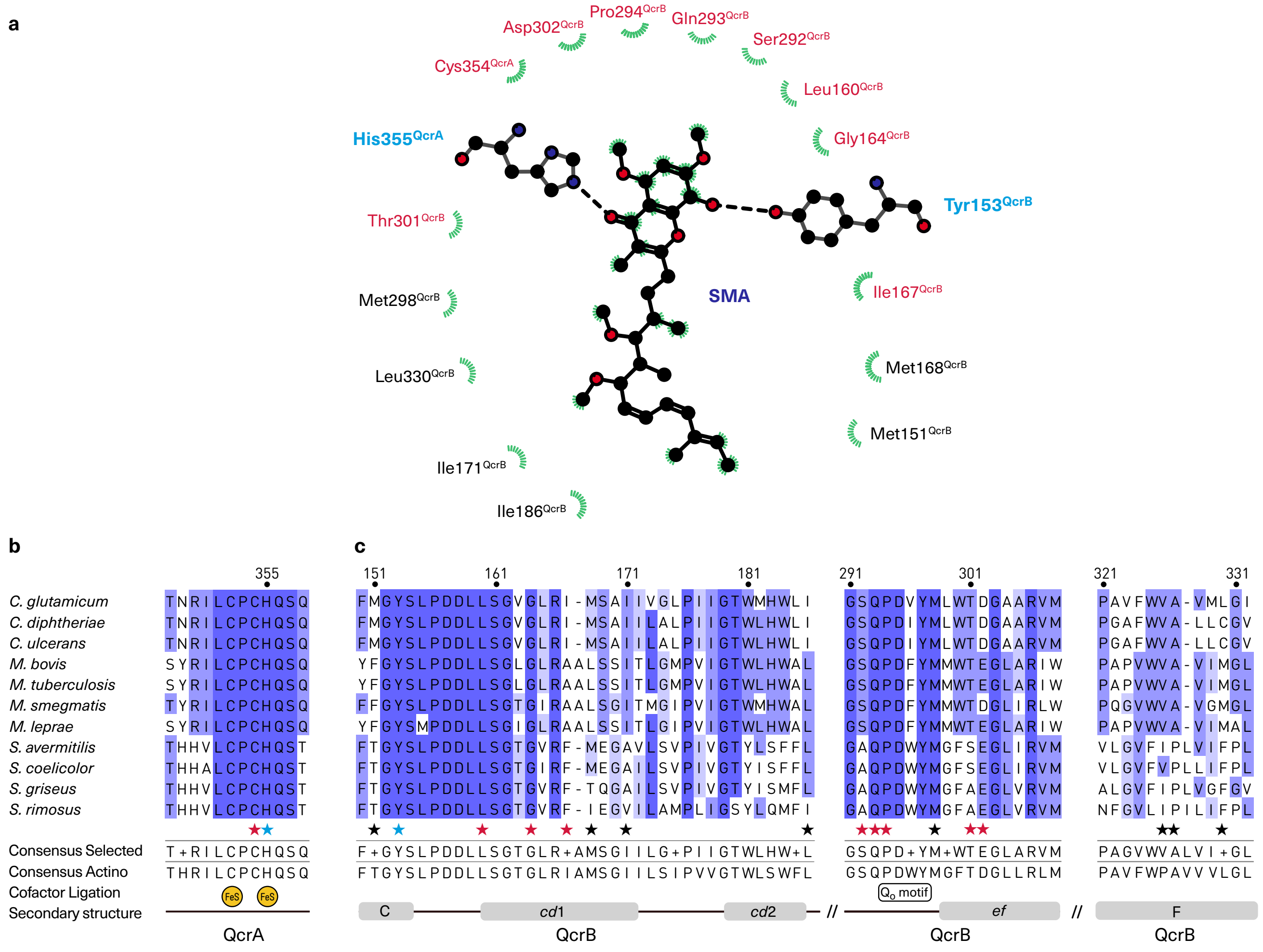

Supplementary Fig. 8 Binding of stigmatellin in the Q_o site and conservation of Q_o site residues.
a Residues are shown that provide direct hydrogen bonds (dotted lines) and non-bonded interactions (green dashed lines) up to 4-Å distance (blue: hydrogen bonding partners of SMA; magenta: residues which interact with the chromone ring derivative, others in black). **b, c** Multiple sequence alignment of QcrA (**b**) and QcrB (**c**) from *C. glutamicum* comparing with selected pathogenic and antibiotics-producing actinobacterial species. The full-length amino acid sequences of QcrA and QcrB were aligned using ClustalOmega (1.2.3) and sections forming the Q_o site are shown. At the bottom, grey boxes indicate transmembrane and surface helices labelled in capitals and italics, respectively. The residues of the Q_o motif [2] are indicated. Amino acid residues which interact with stigmatellin are indicated with a star in the same colour code as in **a**. The consensus sequence of the selected sequences as well as the consensus sequence based on all actinobacterial QcrA or QcrB with 90% identities or lower (to remove identical and highly similar sequences) are shown. NCBI Protein (https://www.ncbi.nlm.nih.gov/protein) sequence accession numbers (QcrA/QcrB): *Corynebacterium glutamicum* ATCC 13032 (NP_601394/ NP_601393), *Corynebacterium diphtheriae* NCTC 13129 (NP_939967/ NP_939966), *Corynebacterium ulcerans* 0102 (YP_006495092/ YP_006495091), *Mycobacterium bovis* AF2122/97 (NP_855867/ NP_855868), *Mycobacterium tuberculosis* H37Rv (YP_006515615/ NP_216712), *Mycobacterium smegmatis* str. MC2 155 (YP_006568914/ YP_888540), *Mycobacterium leprae* TN (NP_301666/ NP_301665), *Streptomyces avermitilis* MA-4680 (NP_827230/ NP_827231), *Streptomyces coelicolor* A3(2) (NP_626405/ NP_626404), *Streptomyces griseus* subsp. griseus NBRC 13350 (YP_001826871/ YP_001826872), *Streptomyces rimosus* subsp. rimosus ATCC 10970 (ZP_20965942/ ZP_20965943).

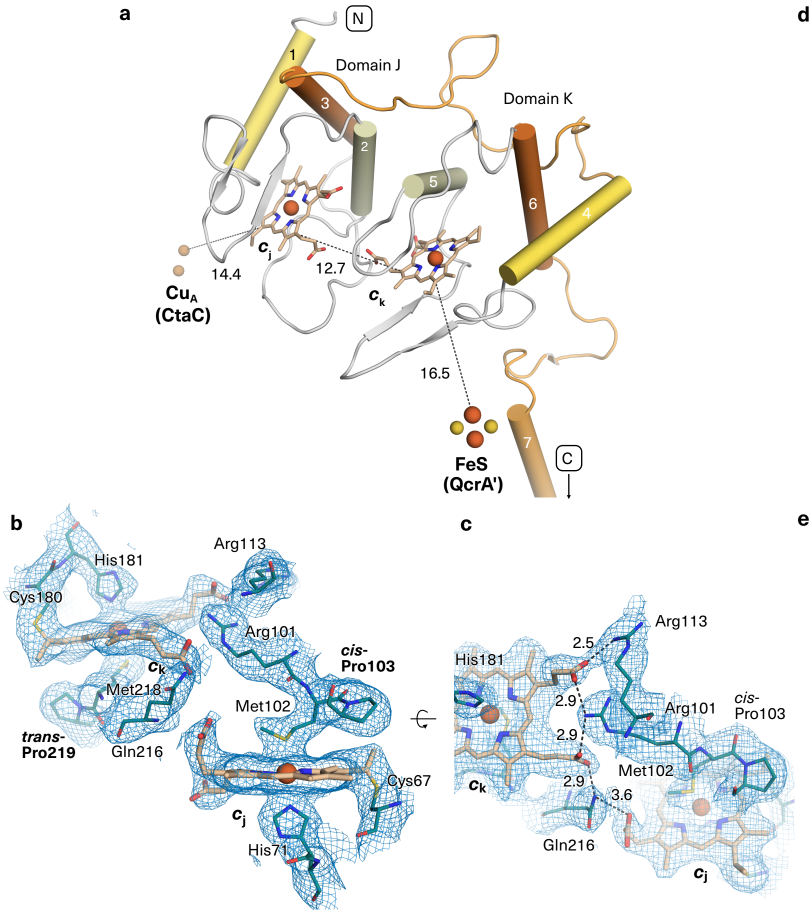

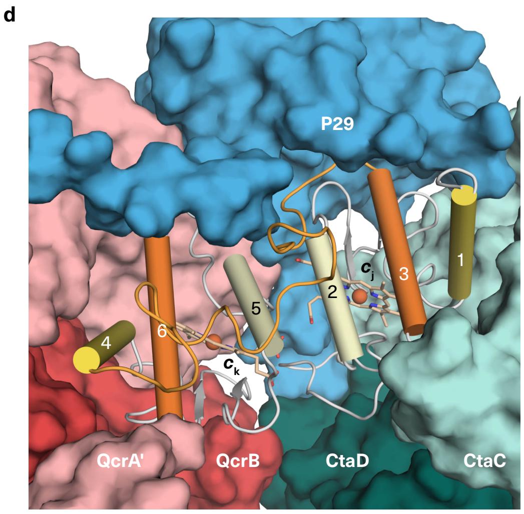

e

Interaction area (Å^2) of QcrC within the supercomplex

	QcrC domains			
	Domain J[1]	Domain K[2]	TMH[3]	QcrC
QcrA'	0	1048	335	1382
QcrB	0	865	1440	2305
P29	1177	659	0	1836
CtaC	1084	0	0	1084
CtaD	436	32	0	468
Sum	2696	2604	1775	7075

[1] Residues 50-138 and haem c_j.
[2] Residues 139-243 and haem c_k.
[3] Residues 244-282.

Supplementary Fig. 9 Structure of QcrC. a Domain composition of domain J composed of three α helices (1, 2, 3) and equivalent helices in domain K (4, 5, 6). The transmembrane helix 7 (truncated in figure) is the C-terminal transmembrane anchor. The edge-to-edge distances (dotted lines) between haem c_j and haem c_k and their electron transfer partners are indicated in Å. Iron atoms: brown spheres, sulfur atoms: yellow spheres. Boxed N and C denotes amino- and carboxy-termini. **b-c** Structure and cryo-EM map (blue mesh) are shown for the environment of haem c_j and haem c_k highlighting the interactions between the domains as well as the *cis*- and *trans*-isomers of the respective proline position in the domains. Hydrogen bonds are depicted as dashed lines and distances are in Å. All residues shown belong to QcrC. **d** Integration of QcrC in the supercomplex. QcrC shown in cartoon presentation with helices as cylinders, other subunits in surface presentation. **e** Interface area (Å^2) of QcrC with adjacent subunits calculated for the entire QcrC, for its individual domains and for the transmembrane helix (TMH) using the PISA server (https://www.ebi.ac.uk/pdbe/pisa/). The boundary between J and K was defined to the middle of the connecting loop. The contour level of all maps was set to 1.8 rmsd.

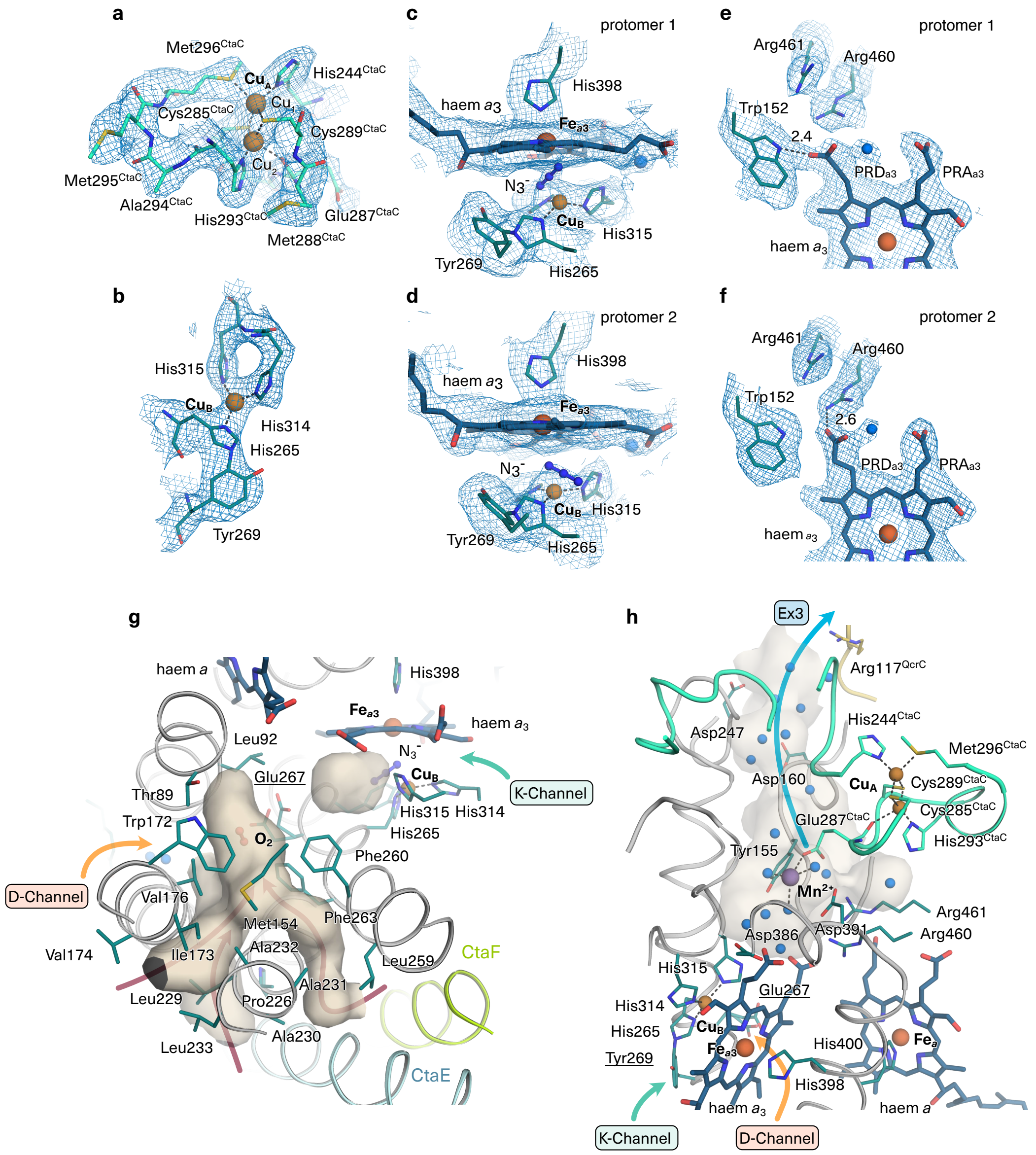

Supplementary Fig. 10 Catalytic centres, cofactors, oxygen delivery and proton exit pathway of cyt *aa*$_3$ oxidase. **a** Coordination (dotted lines) and environment of the di-copper Cu_A site and **b** of Cu_B. All residues belong to CtaD unless noted. **c-d** Azide ions (N_3^-, ball-and-stick representation) resolved in protomer 1 and 2. **e-f** Close-up view of propionate δ (PRD) of haem a_3 in protomer 1 and protomer 2 with hydrogen-bond (dotted line), distances given in Å. **g** Oxygen tunnel (beige) with dioxygen molecule (O_2, ball-and-stick representation) bound, calculated with Glu267 (underlined) in up-conformation as compared to continuous tunnel in down-conformation shown in Fig. 3. The conformation constricts the tunnel above the end of the D- channel leaving a separate small cavity at the catalytic site between Fe_{a3} and Cu_B (marked by an azide ion (N_3^-) in the structure). Residues belong to CtaD (main chain in grey) unless noted CtaD. **h** Proton exit pathway (Ex3) of the oxidase. The colour code is as in **g**. Residues belong to CtaD unless noted. Tyr269 and Gl267 (both underlined) mark the end of K- and D-channel. Water molecules are shown as blue spheres. The grey Ex3 surface was computed using HOLLOW 1.3 with the radius of a water molecule. The contour level of cryo-EM maps (blue mesh) was set to 2.2 rmsd in **a**, 2.0 rmsd in **b**, 1.0 rmsd in **c-d**, 1.8 rmsd in **e**, 1.2 rmsd in **f**.

Supplementary Table 1 | Cryo electron microscopy data collection, structure determination, refinement and validation statistics

	EMDB-13976<br>pdb 7QHM	EMDB-13977<br>pdb 7QHO
**Sample Preparation**		
Protein	*Corynebacterium glutamicum* cytochrome *bcc-aa*$_3$ supercomplex	
Additive 1	Stigmatellin	–
Additive 2	Sodium Azide	–
**Data collection**		
Microscope	FEI Titan Krios with Cs corrector	FEI Titan Krios with Cs corrector
Magnification	75,000	105,000
Voltage (kV)	300	300
Electron exposure (e^{-}Å^{-2})	40.0	59.7
Detector	FEI Falcon3	Gatan K2 Summit with GIF energy filter
Physical pixel size (Å)	0.853	1.08[1]
Defocus range (μm)	-1.0 to -3.0	-1.3 to -3.0
Initial number of particles	431154	73863
Final number of particles for 3D reconstruction	200424	51060
Symmetry imposed	C1	C2
Box size (pixels)	580 $\times$ 580	512 $\times$ 512
Average map resolution (Å)	2.80	3.10
FSC threshold	0.143	0.143
**Refinement**		
Initial model used	*ab initio*	*ab initio*
Model Resolution (Å)	3.0	3.3
FSC threshold	0.5	0.5
Chains	26	26
Atoms	97686 (hydrogens: 48161)	95896 (hydrogens: 47400)
Protein residues	5886	5870
Water molecules	416	10
Ligands	88	78
Model-to-map fit, CC_mask:	0.80	0.76
Bonds (r.m.s.d.)		
Length (Å) (# > 4σ)	0.009 (41)	0.007 (30)
Angles (°) (# > 4σ)	1.082 (56)	0.960 (36)
B-factors (Å^2)		
min/max/mean		
Protein	12.19/73.41/31.99	33.57/81.59/49.70
Ligand	14.31/67.44/30.74	33.97/70.51/48.62
Water molecules	9.43/38.85/23.46	39.88/48.14/44.00
**Validation**		
MolProbity		
MolProbity score	1.24	1.41
Clash score	1.87	4.16
Ramachandran Plot (%):		
Outliers	0.14	0.03
Allowed	4.08	3.30
Favoured	95.78	96.67
Rotamer outliers (%)	0.71	0.46
Cβ outliers (%)	0.00	0.00
Peptide plane:		
cis-proline:	4.6	4.6
cis-general:	0.2	0.1
twisted proline:	0.0	0.0
twisted general:	0.0	0.0

[1] Movies were recorded using the super-resolution mode of 0.54 Å/pixel.

Supplementary Table 2 | Subunit composition of the cryo electron microscopy structure of the *C. glutamicum* *bcc-aa*$_3$ supercomplex.

Corynebacterium glutamicum bcc-aa$_3$ Supercomplex									
Sub-unit	Chain ID	NCBI (UniProt) accession number	Gene name	Range resolved	Matured range	MW[1] kDa	TMH	Cofactors	Functional name
QcrA	A / N	NP_601394 (Q79VE8)	Cgl2190	5–408	1–408	45.2	3	FeS	Rieske ISP
QcrB	B / O	NP_601393 (Q79VE9)	Cgl2189	1–534	1–539	59.8	8	heme b_H , b_L	Cytochrome *b*
QcrC	C / P	NP_601395 (Q8NNK5)	Cgl2191	50-281	49–283[2]	23.7	1	c_j, c_k	Di-heme cytochrome *c*
CtaD	D / Q	NP_601724 (Q9AEL9)	Cgl2523	2–560	1–584	65.1	12	Cu_B, *a*, a_3	Cyt *c* oxidase subunit I
CtaC	E / R	NP_601399 (Q8NNK2)	Cgl2195	29-359	29–359[3]	36.8	2	Cu_A, Mn	Cyt *c* oxidase subunit II
CtaE	F / S	NP_601396 (Q9AEL8)	Cgl2192	16-205	1–205	22.4	5		Cyt *c* oxidase subunit III
CtaF	G / T	NP_601398 (Q8NNK3)	Cgl2194	1-143	1–143	15.5	4		Cyt *c* oxidase subunit IV
P29	H / U	NP_601863 (Q8NMB4)	Cgl2664	32-186	32–194[3]	17.0	0		
P20	I / V	NP_601222 (Q8NP09)	Cgl2017	9-136	1–147	16.4	2		
P12	J / W	NP_599621 (Q8NTD4)	Cgl0373	4-112	1–112	12.1	2		
P8	K / X	NP_600047 (Q8NS61)	Cgl0818	16-73	1–73	8.4	0		
P6	L / Y	NP_599905 (Q8NSJ8)	Cgl0673	3-65	1–65	6.6	2		
ThiX	M/ Z	NP_601532 (P42461)	Cgl2332	23-46	23–190	17.2	0		
Sum						345.4	41		

[1] Molecular weight of the polypeptide is calculated after post-translational processing.
[2] Predicted using the SignalP-5.0 Server with a signal peptide.
[3] Predicted using the SignalP-5.0 Server with a lipoprotein signal peptide.

Supplementary Table 3 | Subunit composition of the cryo electron microscopy structures of the *bcc-aa*$_3$ supercomplex from *Corynebacterium glutamicum* and the homologous supercomplex from *Mycobacterium smegmatis*

C. glutamicum		*M. smegmatis* (pdb 6adq)			*M. smegmatis* (pdb 6hwh)			Identities | Similarities (%)[1]
**Subunit**	**Chain**	**Subunit**	**Chain**	**rmsd**[3]	**Subunit**	**Chain**	**rmsd**[4]	
QcrA	A / N	QcrA	A / M	1.63	QcrA	B / A	1.27	52 | 66
QcrB	B / O	QcrB	B / N	1.26	QcrB	Y / b	1.01	64 | 78
QcrC	C / P	QcrC	C / O	1.08	QcrC	j,K / i,M[5]	1.92[6]	59 | 72
CtaD	D / Q	CtaD	F / R	0.76	CtaD	Q / V	0.63	70 | 84
CtaC	E / R	MSMEG_4268	E / Q	1.18	MSMEG_4268	L / P	1.05	50 | 61
CtaE	F / S	A0R049	G / S	0.80	A0R049	W / Z	0.89	65 | 80
CtaF	G / T	MSMEG_4267	H / T	1.18	MSMEG_4267	S / X	0.99	41 | 55
P29	H / U	LpqE	K / W	4.20	–	–	–	31 | 46
P20	I / V	PRASF1	D / P	1.86	Unknown PolyA	C / G	N/A[7]	38 | 54
P12	J / W	–	–	–	Unknown PolyA	D / H	N/A[7]	–
P8	K / X	–	–	–	Unknown PolyA	F / J	N/A[7]	–
P6	L / Y	CtaJ	I / U	N/A[8]	MSMEG_4693	N / R	N/A[8]	–
ThiX	M / Z	SodC (Fitted)	Y / Z	N/A[8]	–	–	–	–
–	–	MSMEG_4692	J / V	–	MSMEG_4692	O / T	–	–
–	–	–	–	–	Unknown PolyA	E / I	–	–

[1] Sequence identities and similarities are based on NCBI BLAST result of the *C. glutamicum* and *M. smegmatis* protein sequences. Similarities is taken from the positives score from BLAST, which indicate a conservative substitution or substitutions that are often observed in related proteins (https://www.ncbi.nlm.nih.gov/Class/FieldGuide/glossary.html).

[3] The averaged root mean squre deviation (Å) of the *C. glutamicum* model to *M. smegmatis* (pdb 6adq) model of the given chain.

[4] The averagedroot mean squre deviation (Å) of the *C. glutamicum* model to *M. smegmatis* (pdb 6hwh) model of the given chain.

[5] Chain j and i are the two soluble domains of QcrC, chain K and M are their respective transmembrane parts.

[6] Only the rmsd to the soluble domains of pdb 6hwh were calculated.

[7] The respective *C. glutamicum* and *M. smegmatis* subunits occupy roughly at equivalent position with respect to the entire supercomplex but rmsd was not calculated for polyalanine models.

[8] The respective *C. glutamicum* and *M. smegmatis* subunits occupy roughly at equivalent position with respect to the entire supercomplex but neither sequence nor structural homology was found.

Supplementary Table 4 | Mass spectrometry peptide fingerprinting analysis of the *C. glutamicum bcc-aa*$_3$ super-complex.

Sub-unit	Chain ID	Trypsin Uniq Pept	Trypsin Cover (%)	Gluc Uniq Pept	GluC Cover (%)	Chymo Uniq Pept	Chymo Cover (%)
QcrA	A / N	183	82.6	84	56.6	140	79.9
QcrB	B / O	97	40.3	43	23.2	100	51.2
QcrC	C / P	67	60.4	43	68.5	36	63.8
CtaD	D / Q	0	0.0	18	15.8	82	51.0
CtaC	E / R	121	61.6	91	75.5	73	66.2
CtaE	F / S	12	25.9	0	0.0	21	53.4
CtaF	G / T	6	17.5	2	7.7	13	44.1
P29	H / U	38	47.4	31	50.9	30	84.0
P20	I / V	43	51.7	10	19.0	27	47.6
P12	J / W	13	25.9	10	25.4	11	41.1
P8	K / X	25	74.0	24	78.1	26	76.7
P6	L / Y	0	0.0	3	24.6	7	41.5
ThiX	M / Z	3	11.6	21	68.1	19	44.7

* Uniq Pept: number of unique peptides identified; Cover = coverage, the ratio of total length of peptides identified to that of the protein sequence; GluC = endoproteinase GluC; Chymo = chymotrypsin.

Supplementary Table 5 | Degree of sequence conservation (%) of residues in proton transfer pathways of cyt *bcc* complex in Actinobacteria. Unless noted, all residues belong to subunit QcrB. The analysis was performed based on alignments of non-redundant sequences[2]. Values less than 0.5% are not shown.

	Ex1					Ex2			En1	
	**Asp387**	**Arg306**	**Asp302**	**His355[1]**	**Pro294**	**Tyr153**	**Asp295**	**Glu260[2]**	**Glu38**	**Lys253**
Asp	99.5	–	55.9	–	–	–	100	3.2	–	1.9
Glu	–	–	44.1	–	–	–	–	95.8	99.1	0.5
His	–	–	–	100.0	–	–	–	–	–	0.9
Tyr	–	–	–	–	–	99.5	–	–	–	–
Arg	–	99.5	–	–	–	–	–	–	–	–
Lys	–	–	–	–	–	–	–	–	–	94.8
Gln	–	–	–	–	–	0.5	–	–	–	0.5
Asn	–	–	–	–	–	–	–	–	–	0.5
Pro	–	–	–	–	100.0	–	–	–	–	–
Thr	–	–	–	–	–	–	–	–	–	–
Ser	–	–	–	–	–	–	–	–	–	–

[1] QcrA
[2] QcrC

Supplementary Table 6 | Degree of sequence conservation (%) of residues in proton transfer pathways of cyt *aa*$_3$ oxidase in Actinobacteria. Unless noted, all residues belong to subunit CtaD. The analysis was performed based on alignments of non-redundant sequences[1]. Values less than 0.5% are not shown.

	D-channel									K-Channel			
	Glu453[1]	His529	Asp116	Thr36	Asn105	Asn123	Tyr45	Thr97	Glu267	Glu110[2]	Ser280	Lys341	Thr338
Asp	–	–	98.2	–	–	–	–	–	–	–	–	–	–
Glu	97.6	–	–	–	–	–	–	–	98.6	100.0	–	1.1	–
His	–	91.8	–	–	–	–	–	–	–	–	–	–	–
Tyr	–	–	–	–	–	–	98.2	–	–	–	–	–	–
Arg	–	5.0	–	–	–	–	–	–	–	–	–	–	–
Lys	–	–	–	–	–	–	–	–	–	–	–	97.8	–
Gln	0.5	–	–	–	–	–	–	–	–	–	–	0.7	–
Asn	–	0.7	–	–	98.2	97.8	–	–	–	–	–	–	–
Pro	–	–	–	–	–	–	–	–	–	–	–	–	–
Thr	–	–	–	98.2	–	–	–	92.1	–	–	40.9	–	98.2
Ser	–	–	–	–	–	–	–	–	–	–	57.3	–	–

[1] QcrB
[2] CtaC

Supplementary Table 7 | Compositions of D- and K- proton channels and the proton loading site of cyt *aa*$_3$ oxidases of which X-ray or cryo-EM structures are available. The assignment of the constituent residues was referenced to the *Paracoccus denitrificans* structure[3]. Unless noted, all the amino acid residues are in CtaD or COX I (subunit I). The name-giving residues are highlighted in bold typeface. *Corynebacterium glutamicum*: this work; *Mycobacterium smegmatis*: pdb 6adq/ 6hwh; *Paracoccus denitrificans*: pdb 3hb3; *Rodobacter sphaeroides*: pdb 1m56; *Bos taurus*: pdb 5b1a; *Saccharomyces cerevisiae*: pdb 6ymy.

The D-Channel

C. glutamicum		*M. smegmatis*		*P. denitrificans*		*R. sphaeroides*		*B. taurus*		*S. cerevisiae*		Note
His	529	His	528	His	541	His	549	His	503	His	525	
**Asp**	116	**Asp**	115	**Asp**	124	**Asp**	132	**Asp**	91	**Asp**	92	
Thr	36	Thr	35	Thr	26	Thr	24	Thr	10	Thr	11	
Asn	123	Asn	122	Asn	131	Asn	139	Asn	98	Asn	99	
Asn	105	Asn	104	Asn	113	Asn	121	Asn	80	Asn	81	
*Gly	126	Ser	125	Ser	134	Ser	142	Ser	101	Ala	102	
Gly	102	Gly	101	Gly	109	Gly	117	Gly	76	Gly	77	
Thr	97	Thr	96	Ile	104	Ile	112	Met	71	Met	72	
Ser	183	Thr	182	Ser	193	Ser	201	Ser	157	Ser	158	
Glu	267	Glu	266	Glu	278	Glu	286	Glu	242	Glu	243	

[*] Gly126 can be compensated by Ser 186 to establish the equivalent hydrogen bonded network.

The K-Channel

C. glutamicum		*M. smegmatis*		*P. denitrificans*		*R. sphaeroides*		*B. taurus*		*S. cerevisiae*		Note
Glu	110	Glu	95	Glu	78	Glu	101	Glu	62	Glu	82	CtaC/ SU II
Ser	280	Ser	279	Ser	291	Ser	299	Ser	255	Ser	256	
**Lys**	341	**Lys**	340	**Lys**	354	**Lys**	362	**Lys**	319	**Lys**	319	
Tyr	269	Tyr	268	Tyr	280	Tyr	288	Tyr	244	Tyr	245	
Thr	338	Thr	337	Thr	351	Thr	359	Thr	316	Thr	316	

The Proton Loading Site

C. glutamicum		*M. smegmatis*		*P. denitrificans*		*R. sphaeroides*		*B. taurus*		*S. cerevisiae*		Note
His	315	His	314	His	326	His	334	His	291	His	291	
Arg	460	Arg	459	Arg	473	Arg	481	Arg	438	Arg	438	
Arg	461	Arg	460	Arg	474	Arg	482	Arg	439	Arg	439	
Glu	287	Glu	275	Glu	218	Glu	254	Glu	198	Glu	223	CtaC/ SU II
Val	318	Ala	317	Thr	329	Thr	337	Thr	294	Ile	294	
Asp	386	Asp	385	Asp	399	Asp	407	Asp	364	Asp	364	
Asp	256	Asp	244	Asp	193	Asp	229	Asp	173	Asp	198	CtaC/ SU II
Lys	254	Lys	242	Lys	191	227	362	Lys	171	Lys	196	CtaC/ SU II
Phe	387	Phe	386	Arg	400	Arg	408	Leu	365	Val	365	
Ala	390	Thr	389	His	403	His	411	His	368	His	368	

Supplementary References

1. Radu, A. I. *et al*. Toward food analytics: fast estimation of lycopene and β-carotene content in tomatoes based on surface enhanced Raman spectroscopy (SERS). *Analyst* **141**, 4447–4455 (2016).

2. Kao, W.-C. *et al*. The obligate respiratory supercomplex from Actinobacteria. *Biochim. Biophys. Acta* **1857**, 1705–1714 (2016).

3. Koepke, J. *et al*. High resolution crystal structure of *Paracoccus denitrificans* cytochrome *c* oxidase: New insights into the active site and the proton transfer pathways. *Biochim. Biophys. Acta* **1787**, 635–645 (2009).
