# Supplementary material for: Structural basis for safe and efficient energy conversion in a respiratory supercomplex
Source: Nat Commun. 2022 Jan 27;13:545. doi: 10.1038/s41467-022-28179-x (PMC8795186; doi:10.1038/s41467-022-28179-x)
Supplement: Supplementary file 2 — Reporting Summary [file 41467_2022_28179_MOESM2_ESM.pdf]

nature research

Corresponding author(s): Wei-Chun Kao, Carola Hunte

Last updated by author(s): Dec 14, 2021

Reporting Summary

Nature Research wishes to improve the reproducibility of the work that we publish. This form provides structure for consistency and transparency in reporting. For further information on Nature Research policies, see our Editorial Policies and the Editorial Policy Checklist.

Statistics

For all statistical analyses, confirm that the following items are present in the figure legend, table legend, main text, or Methods section.

n/a	Confirmed	
☐	☒	The exact sample size (*n*) for each experimental group/condition, given as a discrete number and unit of measurement
☐	☒	A statement on whether measurements were taken from distinct samples or whether the same sample was measured repeatedly
☒	☐	The statistical test(s) used AND whether they are one- or two-sided<br>*Only common tests should be described solely by name; describe more complex techniques in the Methods section.*
☒	☐	A description of all covariates tested
☒	☐	A description of any assumptions or corrections, such as tests of normality and adjustment for multiple comparisons
☐	☒	A full description of the statistical parameters including central tendency (e.g. means) or other basic estimates (e.g. regression coefficient) AND variation (e.g. standard deviation) or associated estimates of uncertainty (e.g. confidence intervals)
☒	☐	For null hypothesis testing, the test statistic (e.g. *F*, *t*, *r*) with confidence intervals, effect sizes, degrees of freedom and *P* value noted<br>*Give P values as exact values whenever suitable.*
☒	☐	For Bayesian analysis, information on the choice of priors and Markov chain Monte Carlo settings
☒	☐	For hierarchical and complex designs, identification of the appropriate level for tests and full reporting of outcomes
☒	☐	Estimates of effect sizes (e.g. Cohen's *d*, Pearson's *r*), indicating how they were calculated

Our web collection on statistics for biologists contains articles on many of the points above.

Software and code

Policy information about availability of computer code

Data collection	Serial EM 3.6, EPU 2 (Thermo Fisher)
Data analysis	IMOD 4.9.9, cisTEM 1.0.0 beta (including FrealignX), RELION 3.1, SPHIRE 1.3, EMAN2.31, crYOLO 1.5.4, phenix 1.18.2, phenix 1.19.2, phenix 1.20rc1-4392, ChimeraX 1.1, Open-Source PyMol 2.3.0, Coot 0.8.9.2 EL, ISOLDE 1.1, HOLLOW 1.3, RESMAP 1.1.4, ClustalOmega 1.2.3, JalView 2.10.3b1, LIGPLOT 4.5.3, msconvert 3.0.10385, X! Tandem (Version 2017.02.01), PeptideProphet (implemented in TPP v4.7 rev 0, build 201402281256), ProteinProphet (implemented in TPP v4.7 rev 0, build 201402281256), Grade Web Server, 3D FSC Server, SignalP 5.0 server, PISA server.

For manuscripts utilizing custom algorithms or software that are central to the research but not yet described in published literature, software must be made available to editors and reviewers. We strongly encourage code deposition in a community repository (e.g. GitHub). See the Nature Research guidelines for submitting code & software for further information.

Data

Policy information about availability of data

All manuscripts must include a data availability statement. This statement should provide the following information, where applicable:

- Accession codes, unique identifiers, or web links for publicly available datasets
- A list of figures that have associated raw data
- A description of any restrictions on data availability

The cryo-EM density maps are deposited in the Electron Microscopy Data Bank under accession numbers EMD-13976 (SMA-AZI) and EMD-13977 (native). The atomic models of the cryo-EM structures are deposited in the worldwide Protein Data Bank under accession numbers 7QHM (SMA-AZI) and 7QHO (native). The mass spectrometry data are available at MassIVE as MSV000083873 and at ProteomeXchange as PXD014069. The following coordinates from the protein data bank (pdb) were used to set up the restraints for metal-containing cofactor refinement: 1rie, 3cx5, 5b1a. The coordinates of pdb 3cx5 were used for Supplementary Fig.

7h. The coordinates pdb 2a06 and 1be3 were used to calculate the interface area used in Supplementary Fig. 7l. The UniProt (https://www.uniprot.org) Proteome Database was used for LC-MS/MS peptide sequence search. Protein sequences used for multiple sequence alignment for Supplementary Fig. 8 were retrieved from the NCBI Protein Database (https://www.ncbi.nlm.nih.gov/protein).

Field-specific reporting

Please select the one below that is the best fit for your research. If you are not sure, read the appropriate sections before making your selection.

[x] Life sciences [ ] Behavioural & social sciences [ ] Ecological, evolutionary & environmental sciences

For a reference copy of the document with all sections, see nature.com/documents/nr-reporting-summary-flat.pdf

Life sciences study design

All studies must disclose on these points even when the disclosure is negative.

Sample size	For cryo electron microscopic studies, 3453 and 2833 micrograph-movies were recorded respectively for the native and stigmatellin-azide-treated cyt bcc-aa3 supercomplex from Corynebacterium glutamicum. These numbers are sufficient to yield high resolution cryo-EM structures. The number of micrographs used in this study is a typical output of a cryo electron microscopy session using a standard data acquisition scheme using the set-ups detailed in the Methods section.
Data exclusions	The sample size of the electron microscopy experiment was reduced by excluding unsuitable micrographs and particles with the following reasons: Aligned micrographs with excessively low detected fit resolution estimation (worse than 15 Å) and those which CTF failed to be fitted were excluded from further processing. 2D-classification was used to remove false positive particle images such as ice and images which did not contain particles. The initial particle number of the native supercomplex dataset was 73863, and 51060 were used for the final 3D reconstruction. The initial particle number of the stigmatellin-azide-treated supercomplex dataset was 431154, and 200424 were used for the final 3D reconstruction.
Replication	For each cryo-EM structure, eight grids were prepared and screened. The best grid for high-resolution cryo-EM data collection was selected for each structure. Activity assay, menaquinone extraction and quantification as well as SDS-PAGE analysis were performed with four replicates. All replications attempts were successful.
Randomization	Randomization was not relevant to the design of the cryo-EM study. However, randomization was used by programs for some steps of cryo-EM processing: cisTEM ab initio 3D-reconstruction started with random particle images. RELION and cisTEM internally split particles in two random half sets to calculate the Fourier shell correlation for validating the resolution during 3D-refinement. Randomization was not relevant to the design of the biochemical study as no grouping was involved in the experiments.
Blinding	The experimental design did not require blinding for structural and biochemical data, as a defined macromolecular complex was studied.

Reporting for specific materials, systems and methods

We require information from authors about some types of materials, experimental systems and methods used in many studies. Here, indicate whether each material, system or method listed is relevant to your study. If you are not sure if a list item applies to your research, read the appropriate section before selecting a response.

Materials & experimental systems

n/a	Involved in the study	
[x]	[ ]	Antibodies
[x]	[ ]	Eukaryotic cell lines
[x]	[ ]	Palaeontology and archaeology
[x]	[ ]	Animals and other organisms
[x]	[ ]	Human research participants
[x]	[ ]	Clinical data
[x]	[ ]	Dual use research of concern

Methods

n/a	Involved in the study	
[x]	[ ]	ChIP-seq
[x]	[ ]	Flow cytometry
[x]	[ ]	MRI-based neuroimaging
